# *Trypanosoma cruzi*, Chagas disease and cancer: putting together the pieces of a complex puzzle

**DOI:** 10.3389/fcell.2023.1260423

**Published:** 2023-12-21

**Authors:** Cintia Daniela Kaufman, Cecilia Farré, Lucía Biscari, Ana Rosa Pérez, Andrés Alloatti

**Affiliations:** ^1^ Instituto de Inmunología Clínica y Experimental de Rosario (IDICER), Consejo Nacional de Investigaciones Científicas y Técnicas (CONICET), Universidad Nacional de Rosario, Rosario, Argentina; ^2^ Centro de Investigación y Producción de Reactivos Biológicos, Facultad de Ciencias Médicas, Universidad Nacional de Rosario, Rosario, Argentina

**Keywords:** *Trypanosoma cruzi*, cancer, immunotherapy, Chagas disease, microbe-based vaccine

## Abstract

Considering the extensive and widespread impact on individuals, cancer can presently be categorized as a pandemic. In many instances, the development of tumors has been linked to endemic microbe infections. Among parasitic infections, *Trypanosoma cruzi* stands out as one of the most extensively discussed protozoans in the literature that explores the association between diseases of parasite origin and cancer. However, the effective association remains an unsolved paradox. Both the parasite, along with protozoan-derived molecules, and the associated antiparasitic immune response can induce alterations in various host cell pathways, leading to modifications in cell cycle, metabolism, glycosylation, DNA mutations, or changes in neuronal signaling. Furthermore, the presence of the parasite can trigger cell death or a senescent phenotype and modulate the immune system, the metastatic cascade, and the formation of new blood vessels. The interaction among the parasite (and its molecules), the host, and cancer undoubtedly encompasses various mechanisms that operate differentially depending on the context. Remarkably, contrary to expectations, the evidence tilts the balance toward inhibiting tumor growth or resisting tumor development. This effect is primarily observed in malignant cells, rather than normal cells, indicating a selective or specific component. Nevertheless, nonspecific bystander mechanisms, such as *T. cruzi*’s adjuvancy or the presence of proinflammatory cytokines, may also play a significant role in this phenomenon. This work aims to elucidate this complex scenario by synthesizing the main findings presented in the literature and by proposing new questions and answers, thereby adding pieces to this challenging puzzle.

## 1 Introduction

The global burden of cancer is escalating, with its prevalence transforming it into a worldwide pandemic (Sosoniuk-Roche et al., 2020; Sung et al., 2021). Unlike pathologies of infectious origin, the diseases encompassed by the term “cancer” involve agents of illness that are the host’s own cells. These cells have lost replication control and have, to some extent, transformed into pathogenic entities due to the acquisition of a set of functional capacities (Hausman, 2019; Hanahan, 2022).

Several features at the host-parasite interface bear resemblance to those observed at the host-tumor interface. This resemblance allows us to consider neoplastic cells as molecular parasites within their tumor microenvironment (TM). Genes, especially mutations and epigenetic modifications, play a pivotal role in cancer development (Kareva et al., 2015; Narasimhan et al., 2018; Hausman, 2019; Ilango et al., 2020). It is probable that due to these shared characteristics, parasites could contribute negatively or positively to cancer development, respectively. *Trypanosoma cruzi* is frequently mentioned in the literature when analyzing the connection between parasitic diseases and cancer (Yousefi Darani and Yousefi, 2012; Yousofi Darani et al., 2016; van Tong et al., 2017; Callejas et al., 2018; Oliveira et al., 2022; Çelik and Şimşek, 2022).

Nevertheless, to date, the effective association between *T. cruzi*, certain parasite molecules, Chagas disease (CD) resulting from the infection, and cancer remains an enigma. This study aims to elucidate this intricate scenario by integrating the primary findings from the literature while also introducing new questions and answers, thereby contributing to the resolution of this challenging puzzle.

### 1.1 Understanding *Trypanosoma cruzi*

*T. cruzi*, the etiological agent of Chagas disease (CD), is a protozoan characterized by extensive genetic, biochemical, and biological diversity. Consequently, numerous strains exhibit significant differences in terms of pathogenicity, virulence, clinical manifestations, and responses to therapy (Pérez-Molina and Molina, 2018; De Fuentes-Vicente et al., 2019; Zingales and Bartholomeu, 2021).

**FIGURE 1:**
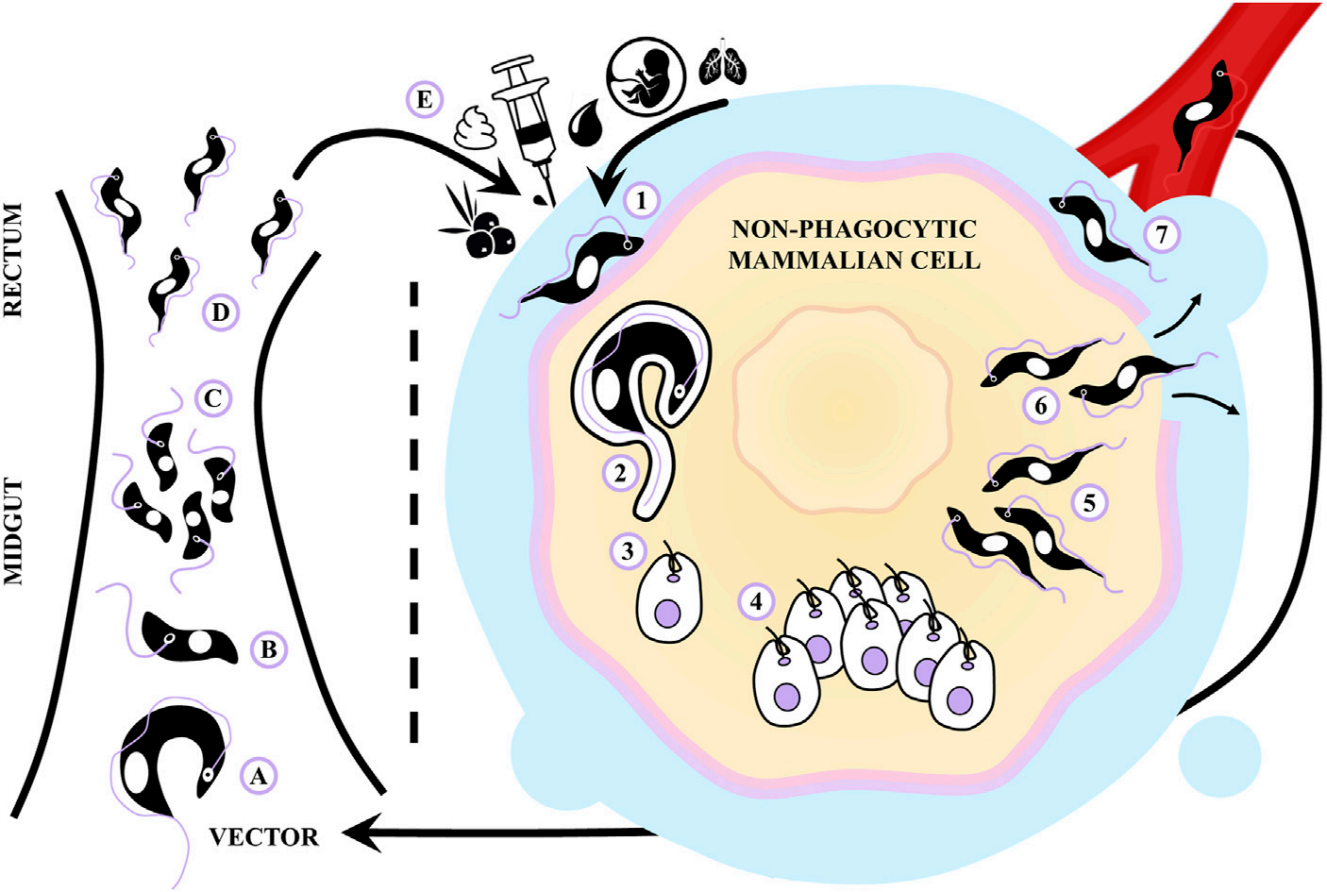
Life cycle of *Trypanosoma cruzi* and routes of transmission. **(A)** The hematophagous triatomine feeds on an infected mammal and ingests blood trypomastigotes. **(B,C)** The non-infectious replicative epimastigote form prevails in the midgut of the insect, **(D)** while in the rectum it transforms into an infectious non-replicative metacyclic trypomastigote. **(E)** When the infected vector feeds on a mammal, it excretes metacyclic trypomastigotes that access to the host through mucous membranes or skin wounds. The parasite can also be transmitted by consumption of contaminated food, blood transfusions, laboratory accidents, organ transplants and from mother to newborns. (1) Once inside the mammalian host, the trypomastigotes are capable of establishing infection in almost all nucleated cells. (2) To promote their entry into non-phagocytic cells, trypomastigotes *T. cruzi* activate host cell signaling pathways involved in the formation of a parasitophorous vacuole. (3) In this vacuole, trypomastigotes receive signals to differentiate into amastigotes and (4) the parasites proliferate as amastigotes in the cytosol. (5) After several cycles of binary division, the amastigotes transform into blood trypomastigotes, (6) which are released from the host cell after lysis, and (7) access to the bloodstream and lymphatic vessels to invade other cells.

This parasite undergoes a complex biphasic life cycle, wherein four distinct cellular forms alternate between two different hosts (Kessler et al., 2017) (see Figure 1). The primary mode of transmission for this protozoan in endemic areas is through the vector route (Pérez-Molina and Molina, 2018). A part of *T. cruzi*’s life cycle involves its passage through the digestive tract of a hematophagous triatomine insect. In the insect’s intestine, the non-infectious replicative epimastigote form predominates, while in the rectum, the parasite undergoes a transformation into the infectious non-replicative metacyclic trypomastigote form. This transformation process involves a differentiation step that allows *T. cruzi* to invade the mammalian host and survive in this new environment (Ramírez-Toloza et al., 2020).

When the vector feeds on a mammal, it excretes metacyclic trypomastigotes that gain access to the host through skin or mucous membrane wounds (Buscaglia et al., 2006). Once inside, these trypomastigotes can establish infection within nearly all nucleated cells, including phagocytes, cardiomyocytes, smooth and striated muscle cells, endothelial cells, adipocytes, and neurons. To facilitate invasion, trypomastigotes trigger various signaling pathways within the host cell, leading to the formation of a parasitophorous vacuole (Costales et al., 2009). Within this vacuole, trypomastigotes sense differentiation signals, escape the vacuole, differentiate into the amastigote form and proliferate in the cytosol. After several cycles of binary division, numerous intracellular parasites can be found in the cell cytoplasm. Eventually, these amastigotes differentiate into blood trypomastigotes, which are released following cell lysis to enter the bloodstream and lymphatic vessels, allowing them to invade other cells (Costales et al., 2009; Li et al., 2016b).

*T. cruzi* can also be orally transmitted through the ingestion of raw or uncooked food or juices contaminated with triatomine feces. In this case, trypomastigotes invade the gastric mucosal epithelium and utilize this entry point to establish a systemic infection (Yoshida, 2008). Additionally, other routes of infection include blood transfusions, organ transplantation, vertical transmission from mother to child during pregnancy, research laboratory accidents, and potentially, sexual intercourse (Bonney, 2014; Gomes et al., 2019).

### 1.2 Chagas disease: etiology, progression, and symptomatology

CD is a neglected infectious threat associated with the persistence of *T. cruzi* in host tissues, influenced by the direct actions of the parasite, the associated inflammatory response, and potential autoimmunity (Kraus et al., 2009; Nagajyothi et al., 2012; Pérez-Molina and Molina, 2018). This disease encompasses two distinct stages. Typically, human infection starts with an acute phase characterized by high parasitemia and cellular parasitism, lasting up to 2 months. This phase may be asymptomatic or exhibit non-specific symptoms, including fever, fatigue, lymphadenopathy, splenomegaly, hepatomegaly, gastrointestinal manifestations, and inflammation at the site of inoculation. In very rare instances, myocarditis, encephalitis, or meningoencephalitis can lead to fatal outcomes during this stage (Pérez-Molina and Molina, 2018; Munari et al., 2019).

During the acute phase, a relatively efficient host immune response correlates with a reduction in parasitemia, but does not completely clear the infection. Consequently, *T. cruzi* can persist into the chronic phase of the disease (Nagajyothi et al., 2012). Chronic patients can remain asymptomatic for decades, a stage known as the indeterminate phase, and their diagnosis is typically confirmed through *T. cruzi* antibody-specific tests (Vargas-Zambrano et al., 2013). Regrettably, 30% of patients will experience organ dysfunction, characterized by cardiac, gastrointestinal (megacolon and megaesophagus), and/or peripheral nerve lesions (Vargas-Zambrano et al., 2013; Chadalawada et al., 2020).

The reasons why some individuals remain asymptomatic while others experience disease progression to a fatal outcome remain unclear. The mechanistic details behind the various clinical patterns exhibited by CD have yet to be fully elucidated. Nonetheless, the balance between infection persistence and the host immune response appears to be a critical factor, with inflammation potentially playing a central role. Other factors contributing to heterogeneity include the diverse lineages of the protozoan, its virulence and tissue tropism, and host characteristics such as the nature of the antiparasitic immune response and genetic factors (Costales et al., 2009; Nagajyothi et al., 2012; Ramírez-Toloza et al., 2020; Magalhães et al., 2022).

### 1.3 *T. cruzi*, Chagas disease, and cancer: the paradox unveiled

The differential cellular tropism of *T. cruzi*, indicating a biological specificity or preference for particular organs, prompted Roskin to investigate whether tumors possess the ability to selectively recruit this parasite over other host cells (Krementsov, 2009). The discovery of mutual inhibition between implanted tumors and the infection in animal models marked the beginning of the paradoxical narrative involving *T. cruzi*, CD, and cancer (Roskin and Exempliarskaia, 1931).

The anticancer activity of experimental *T. cruzi* infection, observed in both acute and chronic phases, and the lysates of parasite trypomastigotes and epimastigotes was documented in various experimental models encompassing mice (across different strains), rats, guinea pigs, and rabbits, using various virulent and avirulent parasite strains. At that time, this phenomenon was characterized as the interaction between two processes (CD and malignant growth), two organisms (the parasite and the host), and two cell types (the protozoan and the neoplastic cell) (Kallinikova et al., 2001). Essentially, it was described as “a complex process of struggle between cancer cells and trypanosomes” which was hypothesized to potentially occur in the context of human cancer (Roskin and Exempliarskaia, 1931).

Hence, the subsequent investigation focused on exploring the antitumor potential of parasite extracts in humans. These experiments could be performed due to the absence of stringent ethical regulations. Commercial preparations, specifically known as Cruzin/KR vaccine and Trypanosa, were scrutinized for their demonstrated specific, direct, and dose-dependent inhibition of neoplastic growth. These effects were observed both *in vitro* with human malignant cells and *in vivo* in experimental models (Roskin and Exempliarskaia, 1931; Kallinikova, 1964; Kallinikova and Kats, 1968; Kats, 1968; Leikina, 1968; Leikina and Smirnova, 1968; Levinson et al., 1968; Roskin, Kallinikova, et al., 1968; Roskin and Ogloblina, 1968; Roskin and Sokolova, 1968; Roskin, Struve, et al., 1968). These compounds exhibited therapeutic effects, reducing tumor volume to a surgical size, achieving complete remission of neoplasms, and demonstrating analgesic effects, as well as reduced inflammation and bleeding (Bongard and Roskin, 1939; Klyueva, 1946).

In the early 1950s, the effects of injecting live parasites into human patients to control tumor progression were examined. In these instances, although tumor size and pain were reduced, the survival rate was not significantly higher (Galliard et al., 1950).

However, the paradox became more pronounced when not only a lack of effect but also observed toxicity and pro-tumor activities with *T. cruzi* preparations were documented (Lob, 1949; 1950). The anticancer effect of the parasite lysates was consistently less reliable than that of the protozoan infection. This discrepancy might be attributed to the lack of reproducibility in parasite isolation and preparation (Kallinikova et al., 2001).

This apparent contradiction gained significance when a higher frequency of certain malignant neoplasms was observed in patients with CD compared to healthy individuals (Chapadeiro et al., 1964). In endemic areas, the analysis of CD prevalence in cancer patients revealed a notable rate of *T. cruzi* seropositivity (Davila Rosenthal et al., 2016). Furthermore, another study found that only one-third of deaths in CD patients were directly related to the disease, with neoplasms being the leading cause of death (Ramos-Rincon et al., 2021). Interestingly, a related study on CD mortality by Santo (2009) identified neoplasms as one of the primary underlying causes of death, although a comparative analysis with healthy individuals was absent.

Additionally, other studies have reported that the leading causes of death in older CD patients are commonly associated with heart disease rather than cancer (de Menezes et al., 1989). While no specific association between CD and particular tumors has been reported in the clinical setting, a correlation between cancer and megavisceral manifestations has been noted (discussed in Section 1.5) (Garcia et al., 2003; Gullo et al., 2012). In this complex scenario, where evidence points to both antineoplastic and pro-tumorigenic properties of *T. cruzi*, it is evident that the interaction of the parasite, its molecules, the host, and cancer involves diverse mechanisms that operate differentially depending on the observational context. The outcome of this intricate interaction depends on various factors: 1) the type of tumor, 2) the virulence and phenotypic characteristics of the *T. cruzi* strain involved—for instance, some clones exhibit antitumor effects only during infection or immunization, while others exhibit a delayed effect—(Batmonkh et al., 2006), 3) whether the immunogens are parasite lysates or live parasites, and 4) in the case of live parasites, the stage of the disease—acute or chronic phase—regardless of the infected host species.

The pro-tumoral role of *T. cruzi* has been predominantly associated with the parasite’s capacity to act as a carcinogen, since persistent infection with consequent inflammation damages the DNA and produces changes in gene expression, and immunosuppression of the host (Plata, 1985; Dominical et al., 2010). On the other hand, the antitumor effects of *T*. *cruzi* and CD encompass a wide range of possibilities, including the hypothetical existence of a toxin with destructive effects on neoplastic cells, the selective invasion and destruction of abnormal cells by trypanosomes (Krementsov, 2009), interference with the metabolism of infected cells (Li et al., 2016b), alterations in neuronal signaling (Kannen et al., 2015), and stimulation of a cross-specific or non-specific immune response through lysates and infection (Melnikov et al., 2004).

### 1.4 *T. cruzi* and the hallmarks of cancer

Cancer is a complex and progressive disease driven by gene-environment interactions, requiring dysfunction in multiple systems. It is evident that *T. cruzi* infection or its components may have a direct or indirect impact on these systems (Knox, 2010).

**FIGURE 2:**
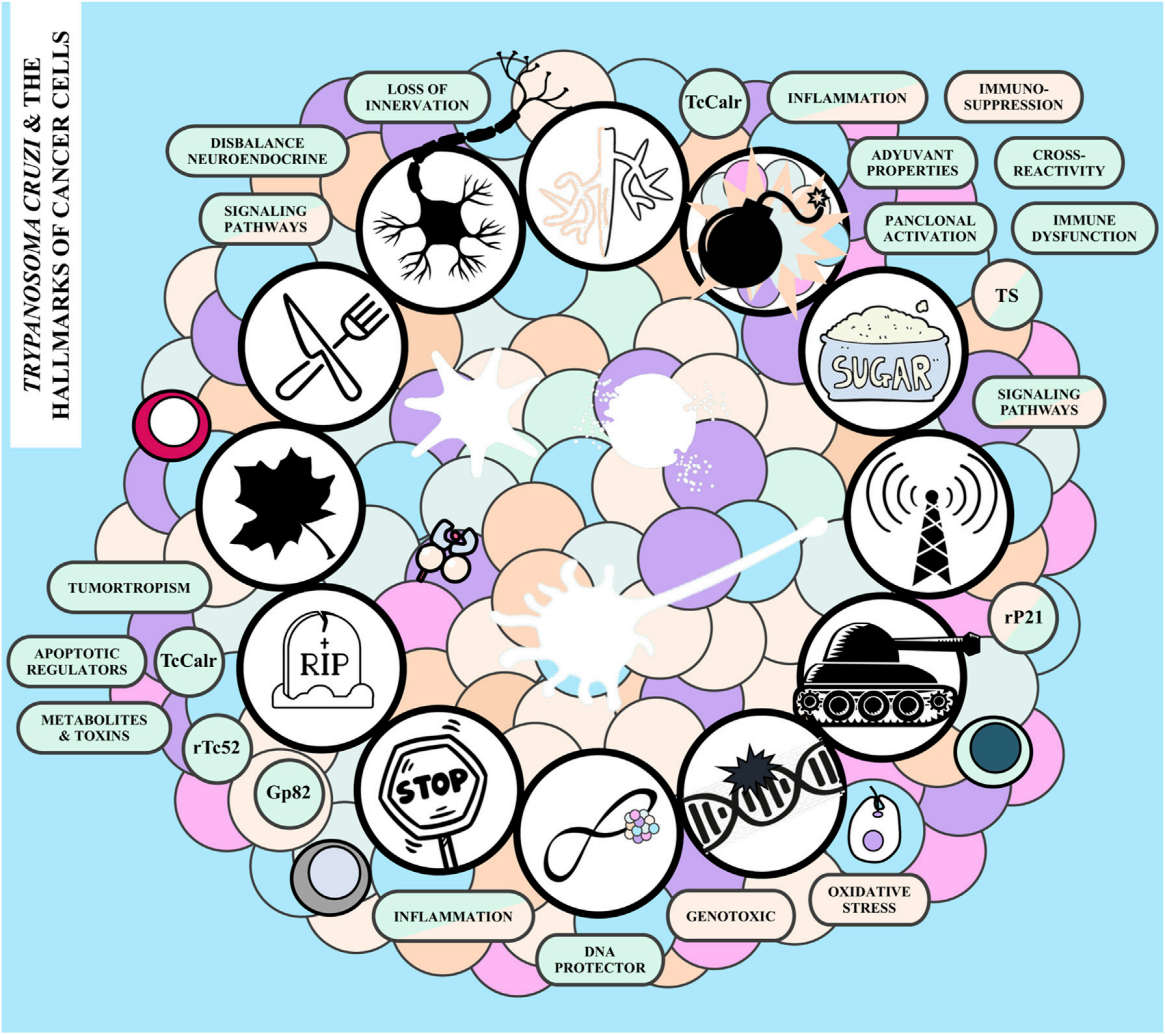
*Trypanosoma cruzi* and the hallmarks of cancer. The hallmarks of cancer following the hands of the clock starting from the top: inducing vasculature, avoiding immune destruction, exhibiting aberrant glycosylation patterns, sustaining proliferative signaling, activating invasion and metastasis, promoting genomic instability, enabling replicative immortality, resistance to growth suppressors, resisting cell death, stimulating cellular senescence, reprogramming energy metabolism, and altering neuronal signaling. The boxes adjacent to each hallmark summarize the potential pro-tumor (light blue), anti-tumor (pink) and mechanisms that could operate in both directions (pink and light blue) involved. TS, *T. cruzi trans*-sialidase; Gp52, *T. cruzi* Gp82 protein; rP21, *T. cruzi* P21 recombinant protein; TcCalr, *T. cruzi* Calreticulin, rTc52, *T. cruzi* protein 52.

For a healthy cell to transform into a cancer cell and subsequently proliferate into a tumor, it must undergo a series of intricate changes. These changes occur through diverse mechanisms and at various stages, leading to the acquisition of specific “enabling characteristics” that propel “functional traits”. These traits are collectively referred to as “The Hallmarks of Cancer” (Hanahan and Weinberg, 2011; Hornsveld and Dansen, 2016). Elements such as genomic instability, inflammation, aberrant glycosylation, neuronal alterations, and cellular senescence, which belong to the first class of hallmarks, promote the survival, proliferation, and spread of neoplastic cells. On the other hand, the acquired capabilities include sustaining proliferative signaling, evading growth suppressors, resisting cell death, enabling replicative immortality, inducing vasculature, activating invasion and metastasis, reprogramming cellular metabolism, and avoiding immune destruction (Munkley and Elliott, 2016; Senga and Grose, 2021; Hanahan, 2022). There is experimental evidence to sustain that *T. cruzi* can affect the tumor growth at different levels (Figure 2). In the following sections we aim to summarize how, when and to which extent the parasite -and its associated molecules-interact with each of the aforementioned processes or hallmarks, imprinting a particular outcome that may favor tumor growth, or tumor control.

#### 1.4.1 Inflammation and avoidance of immune destruction

The majority of cells that constitute tumors are not malignant or aberrant but rather consist of healthy cells recruited by neoplasms, including inflammatory cells, which actively participate in tumorigenesis (Hausman, 2019).

Inflammation serves as an effective defense mechanism against infection and injury, yet unresolved or chronic inflammation acts as a driving force for carcinogenesis (Chen et al., 2018; Zhao et al., 2021). Notably, the inflammatory process can promote the transformation of an incipient tumor into cancer by supplying growth, survival, and pro-angiogenic factors. Inflammation also recruits enzymes that modify the extracellular matrix, promoting angiogenesis, invasion, metastasis, and DNA damage, associated with mutations, malignancy, and evasion of host defense mechanisms (Hanahan and Weinberg, 2011).

Infection by *T. cruzi* triggers multiple immune effectors, including pro-inflammatory cytokines, lytic antibodies, and the concerted activities of Natural Killer (NK) cells, phagocytes, helper, and cytotoxic T lymphocytes (HTL and CTL, respectively) (Rios et al., 2019). This robust immunity, persistent throughout the infection, prevents the recurrence of intense parasitism and secondary infection. This immunity may have pro- or anti-tumor effects by altering the TM (Junqueira et al., 2011; Jiang et al., 2022).

However, the establishment of CD also depends on a favorable environment for intracellular parasite proliferation, avoiding immune elimination (Chain et al., 2020). The persistence of the protozoan in the indeterminate and chronic phases yields antigens (Ags) and Pattern Recognition Receptor (PRR) ligands that induce extensive localized inflammatory reactions, characterized by macrophage and T lymphocyte (TL) infiltration, contributing to the pathogenesis of the disease (Galili, 2013; Macaluso et al., 2023). This has been associated with immune cell dysfunction (Duran-Rehbein et al., 2017). It is well known that *T. cruzi* induces a state of nonspecific immunosuppression in the host, especially during the acute phase (Müller et al., 2018). Parasite-derived factors could induce immune cell apoptosis to promote parasite survival and growth (Ouaissi and Ouaissi, 2005), which may explain the increased tumor incidence observed in some animal models (Plata et al., 1986).

**FIGURE 3:**
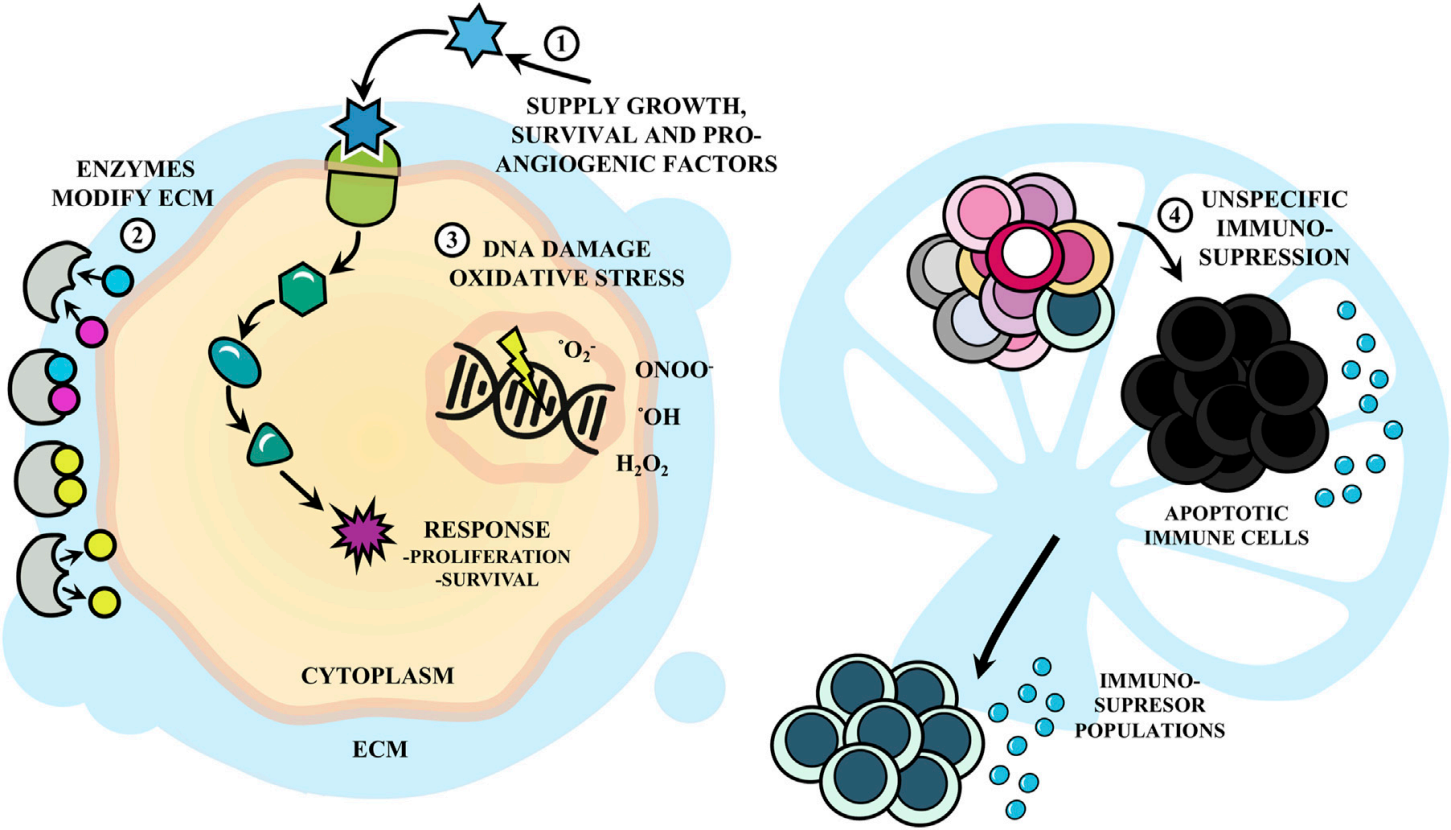
Inflammation in response to *Trypanosoma cruzi* infection as a driving force for cancer. The inflammatory response following infection with *T. cruzi* can (1) supply growth, survival and pro-angiogenic factors to tumor cells, as well as (2) enzymes that modify the extracellular matrix (ECM), and (3) generate DNA damage by oxidative stress. These promote proliferation, survival, invasion and malignancy. Also, (4) the infection promotes immunosuppression with apoptosis of immune cells.

**FIGURE 4:**
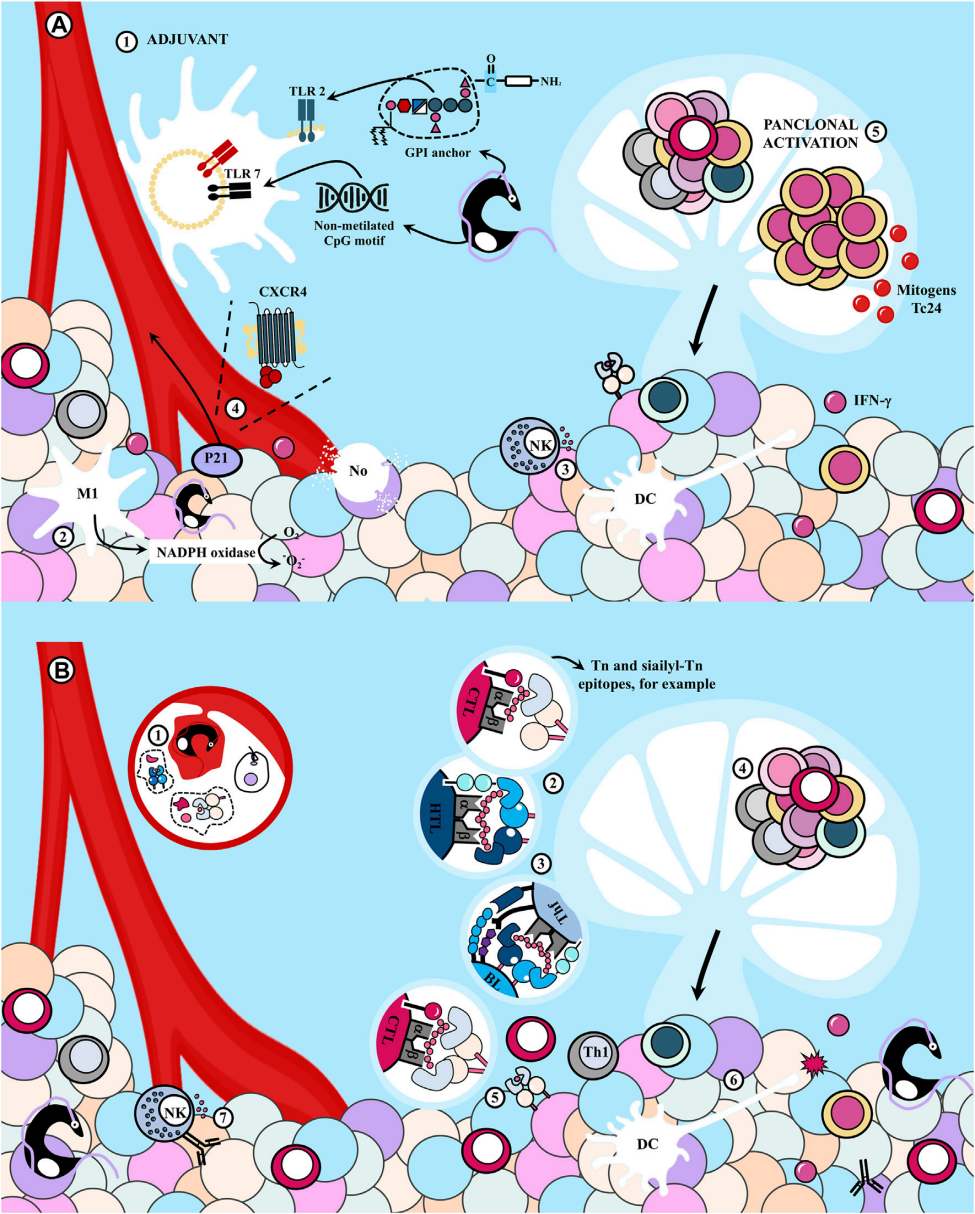
Antitumor effects of the immune response elicited during *Trypanosoma cruzi* infection. **(A)** (1) *T*. *cruzi* as an adjuvant enhances tumor immunogenicity because it presents potent agonists of Toll-like receptors (TLR), including glycosylphosphatidylinositol (GPI) anchors and unmethylated CpG motifs. (2) This induces a strong innate response involving macrophages -probably of M1 phenotype- and dendritic cells (DC), with strong NADPH oxidase activity which could mediate tumor cell killing, in addition to (3) Natural Killer (NK) cells. Also, the parasite (4) releases the P21 protein that attracts leukocytes interacting with CXCR4 and (5) molecules that could act as mitogens inducing polyclonal responses of B and T lymphocytes (BL and TL, respectively), eventually promoting the recognition of cancer cells. **(B)** Infection with *T. cruzi* induces a cross-immune response against tumor antigens (Ags) that were previously expressed on the parasite. Thus, (1) the protozoan could infect DC or the DC could internalize parasite Ags and (2) present parasite epitopes to naïve TL in the secondary lymphoid organs (SLOs). (4) Those able to recognize the Ags proliferate and differentiate into effector and memory populations. BL can recognize conformational Ags and acquire stimulatory signals from follicular T helper (Tfh) lymphocytes. (4) Cytotoxic effector T lymphocytes (CTL) leave the SLOs (5) to eliminate cancer cells if they are able to recognize tumor epitopes by cross-reactivity, while type helper type 1 (Th1) cells secrete cytokines that contribute to the antitumor potential. (6) DC uptake tumor Ags from lysis of neoplastic cells and promote the antitumor immunity. (7) Cross-reactive antibodies produced by BL could have antineoplastic effects by antibody-dependent cellular cytotoxicity (ADCC). M1, macrophage of M1 phenotype; No, neutrophil.

Additionally, apart from the pro-tumoral mechanisms described in Figure 3, it has been suggested that both infection and immunization with parasite extracts exert antitumor effects, at least partially, by activating various components of the immune system (Morillo et al., 2014; Ubillos et al., 2016; Freire et al., 2022). This hypothesis finds support in the observation that these effects are absent when host immune defenses are suppressed (Roskin and Romanova, 1935; 1936a; 1936b; 1937; 1938a; 1938b). Several hypotheses have been proposed in this regard, as summarized in Figure 4.

##### 1.4.1.1 *T. cruzi* as an adjuvant for antitumor immunity

There is a significant correlation between the oncoprotective effect and the interval between tumor inoculation and *T. cruzi* infection. Early infection in relation to tumor challenge results in smaller tumors and earlier regression (Kallinikova, 1964). Hence, the innate arm of the immune system appears to be crucial.

*T. cruzi* acts as an adjuvant, enhancing tumor immunogenicity and generating a potent immune response that drives an efficient antitumor response. The parasite activates the host’s innate immune response by triggering Toll-like receptors (TLR), efficient detectors of both pathogens and cancer cells, promoting innate cell activation and contributing to TLs priming (Morillo et al., 2014). Intrinsic TLR agonists in the parasite, such as glycosylphosphatidylinositol (GPI) anchors and unmethylated CpG DNA and RNA motifs, continuously stimulate Th1 lymphocytes (Junqueira et al., 2012).

Altered expression of TLR 2, 4, and 9 during infection has been reported, leading to an exacerbated inflammatory response that promotes tumor cytolysis (Morillo et al., 2014). The immunostimulatory effect results in elevated levels of proinflammatory cytokines that activate effector mechanisms, efficiently destroying neoplastic cells. For example, IFN-γ, found in high levels in tumors of infected mice, can induce cell cycle arrest and dormancy in cancer cells, and stimulate NK cell activity (Freire et al., 2022). Junqueira et al. (2012) employed *T. cruzi*-derived unmethylated CpG motifs as adjuvants in a vaccine carrying the NY-ESO-1 tumor antigen (TAg), leading to a significant delay in the growth of B16-F10 tumors expressing NY-ESO-1, associated with the magnitude of the T response elicited by the adjuvant (Junqueira et al., 2012). Immunizations with an epimastigote lysate also protected prophylactically and therapeutically by inducing antitumor immune responses against the LL/2 tumor. The effect likely involves ligands of TLR and essential mechanisms for the early host response against the protozoan (Freire et al., 2022). Likewise, Ubillos et al. (2016) observed that vaccination with parasite lysates significantly inhibited tumor development in rats by inducing an innate response involving an increased number of macrophages, likely of M1 phenotype, and dendritic cells (DCs) with potent NADPH oxidase activity, which could mediate tumor cell destruction. Additionally, Zenina et al. (2008) found tumor growth inhibition in animals previously immunized with *T. cruzi* trypomastigote lysates, an effect associated with the nonspecific stimulation of NK cells.

Notably, the parasite’s ability to persist in host tissues and induce a long-term Ags-specific immune response, coupled with intrinsic TLR agonists, *T. cruzi*’s replication in the host cell cytoplasm stimulating CTL, positions *T. cruzi* as not only an intriguing immunological adjuvant but also a potential vaccine vector. A live, recombinant attenuated clone of the parasite carrying the NY-ESO-1 TAg, used by the group of Gazzinelli, induced a robust, long-lasting antitumor immune response with a strong innate arm activation, superior to that induced by vaccination with the TAg in combination with other classical TLR agonists (Junqueira et al., 2011).

It should also be noted that *T. cruzi* releases various molecules with other immune system effects. For instance, the P21 protein, with a broad spectrum of biological functions, can attract leukocytes by interacting with CXCR4 (Borges et al., 2020).

##### 1.4.1.2 Panclonal activation of immune cells

During *T. cruzi* infection, several molecules that act as mitogens are released, inducing polyclonal responses in B lymphocytes (BL) and TL. This lack of specificity in the immune response can promote the recognition of cancer cells. For example, Tc24, a flagellar calcium-binding protein of the parasite, serves as a non-specific BL activator. *In vivo* treatment with its recombinant version (rTc24) leads to a rapid increase in the diversity of immunoglobulins secreted by BL independently of TL, and this response is mostly unrelated to parasite Ags in the extracts or to the protein itself, indicating a polyclonal expansion of non-specific BL clones (Ouaissi and Ouaissi, 2005). It is observed that the acquisition of tumor resistance and the host response to the parasite occurs concurrently (Kallinikova et al., 2001).

##### 1.4.1.3 Cross-reactivity between *T. cruzi* and tumors. The molecular mimicry hypothesis

Cross-reactivity between *T. cruzi* and host tissues is a widely debated topic in CD physiopathology. It is suggested that CD is an infection-induced autoimmune disease, wherein the infection disrupts the host’s ability to differentiate between self and foreign (Petry et al., 1988; Teixeira et al., 2011). Though the origins of these autoimmune events have been a matter of controversy, it is proposed that they are induced by the cross-reactivity of Ags present in both the parasite and the host (Pérez-Molina and Molina, 2018). There is key evidence to support the theory of molecular mimicry, with the parasite potentially using this mechanism of “Antigenic disguise” to attenuate the immune response (Ouaissi and Ouaissi, 2005). However, the generation of autoimmunity is likely reliant on the disruption of immune regulation preventing the response to self-Ags (Perl, 2012; Pisetsky, 2023).

The primary argument against this theory is supported by experiments in which the organs typically affected by CD remain undamaged in animals immunized with parasite Ags (Hauschka et al., 1947; Malisoff, 1947; Hauschka and Goodwin, 1948; Spain et al., 1948; Cabral, 2000; Batmonkh et al., 2006). Nevertheless, it has been observed that immunization with *T. cruzi* proteins such as cruzipain, B13, Cha and ribosomal proteins or parasite extracts induce autoimmune events (Leon et al., 2004). Furthermore, TL directed against parasite Ags can recreate nerve tissue pathology when transferred to unexposed animals, and experimental immunization with *T. cruzi* Ags is associated with IgG deposits in cardiac tissue (Giordanengo et al., 2000).

Probably, the observed differences are highly dependent on the dose used of parasite lysates or proteins; however, the existence of: 1) antibodies capable to recognize both parasite and host Ags and, 2) cellular autoimmune events suggests that tumor cells and *T. cruzi* likely present similar (or even identical) epitopes to immune cells (Gironès et al., 2005; Novaes et al., 2016; Ubillos et al., 2016). Thus, the infection likely induces a cross-immune response against TAgs (Zhigunova et al., 2013; Campo et al., 2014).

Parasitic extracts have demonstrated comparable antitumor effects to infection in animal models (Krementsov, 2009). Eligio García et al. (2022) produced polyclonal antibodies against various *T. cruzi* strains in rabbits, observing reactivity against protein extracts from acute lymphoblastic leukemia and neuroblastoma cells. Additionally, these anti-*T. cruzi* antibodies exhibited cross-reactivity with human tumor cell lines, including breast, colon, and cervical cancer, along with human plasmacytoma (Hernandez-Munian et al., 1980; Levin, 1991; Ubillos et al., 2016). Virulent and avirulent *T. cruzi* strains exhibited oncoprotective effects against sarcoma-180 and Ehrlich’s adenocarcinoma, which correlated directly with the antibody titer against the parasite at the time of tumor implantation. This effect was notably amplified at the peak of the immune response. Furthermore, the most immunogenic strains of *T. cruzi* conferred improved oncoprotection (Kallinikova et al., 2006).

Zenina et al. (2008) demonstrated that a trypanosome lysate effectively inhibited the growth of Ehrlich’s adenocarcinoma when administered prophylactically, despite not generating a substantial antibody response. The process involved the cellular component of adaptive immunity, as tumor growth was significantly decelerated when splenocyte transfer was conducted from lysate-immunized animals.

In other cases, both branches of adaptive immunity were engaged. Specific antibodies against *T. cruzi* recognized membrane and intracellular molecules in lung cancer cells (LL/2), inducing cell death via antibody-dependent cellular cytotoxicity (CCDA). Furthermore, splenocytes from mice immunized with a *T. cruzi* lysate produced significantly higher levels of IFN-γ when restimulated with LL/2 tumor lysate. Thus, the parasite extract contained shared molecules with these tumor cells (Freire et al., 2022).

Likewise, vaccination with epimastigote lysates substantially inhibited tumor development in two rat tumor models resembling human colon and breast carcinogenesis. The process was accompanied by the activation of specific HTL and CTL, cellular cytotoxicity, and specific antibodies against both tumors capable of mediating CCDA (Ubillos et al., 2016).

There is mounting evidence suggesting that parasites and tumor cells express similar mucin-like antigenic structures (Osinaga, 2007). Zenina et al. (2008) noted the resemblance between tumor mucins and *T. cruzi* surface glycoproteins, while Freire et al. (2022) found that carbohydrates were essential for inducing the antitumor immune response, with their oxidation diminishing their antineoplastic activity.

Despite the preponderance of *O*-glucans in *T. cruzi* binding to Ser/Thr residues via α-*N*-acetylglucosamine (α-GalNAc), the parasite also expresses tumor-associated Ags, such as Tn and sialyl-Tn Ags, which can induce an effective immune response against neoplastic cells (Freire et al., 2003; Sedlik et al., 2016). Indeed, chronically infected mice treated with 1,2-dimethylhydrazine (DMH), a compound inducing a human-like colon tumor model expressing Tn and sialyl-Tn Ags, exhibited lower malignancy rates than uninfected animals exposed to DMH (Oliveira et al., 2001). This suggests that glycoproteins of the parasite may act as immunogens.

Similarly, mice that received splenocytes from animals immunized with type II or III mucins and were subsequently inoculated with Erlich’s adenocarcinoma experienced reduced tumor growth. Interestingly, the oncoprotection observed did not differ from that found when mice were prophylactically immunized with a trypanosome lysate (Zenina et al., 2008).

Certainly, the pronounced variability of the polysaccharides explains the capacity of *T. cruzi* to evade the host’s immune control and underlies a high probability of coincidence between immune targets in the protozoan’s Ags and the mucins of cancer cells (Zenina et al., 2008).

It is important to note that the suppression of tumor growth observed when tumors were transplanted immediately after infection cannot be explained by adaptive immune mechanisms alone, underscoring the need to consider at least one additional mechanism at play (Melnikov et al., 2004).

Furthermore, akin to *T. cruzi,* which demonstrates antigenic polymorphism and diverse epitopes, cancer cells can also modulate their Ags to evade immune system-mediated destruction. Consequently, the relevance of this mechanism remains uncertain, needing further investigation to clarify this complex scenario.

#### 1.4.2 Genomic instability

Cancer is fundamentally a genetic disease, and the acquisition of most of its defining hallmarks relies significantly on genomic alterations (Hanahan and Weinberg, 2011; Hausman, 2019). Somatic cells give rise to tumor formation and propel tumor progression by progressively accumulating and tolerating oncogenic and tumor suppressor mutations, and occasionally by gaining and losing entire chromosome segments (Andor et al., 2017; Bashyam et al., 2019).

Carcinogenesis is associated with the generation of free radicals and nitric oxide (Maeda, 1998). *T. cruzi* can be considered a genotoxic agent due to its capacity to (a) induce DNA damage through the generation of reactive oxygen species (ROS) and (b) elicit inflammation, subsequently leading to the expression of nitric oxide synthases catalyzing the production of nitric oxide by conversion of L-arginine to citrulline (Wink et al., 1991; Ohshima et al., 1992; Silva et al., 1995; Bergeron and Olivier, 2006; Nakamura et al., 2006).

**FIGURE 5:**
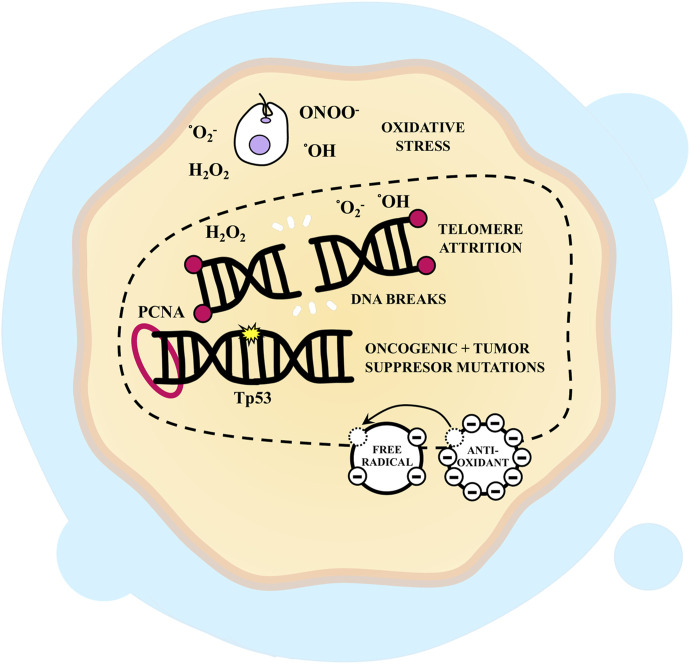
Genomic instability, replicative immortality, evasion of growth suppressor, cellular senescence and *Trypanosoma cruzi*-infected cells. *T. cruzi* can act as a carcinogen and damage host cell DNA by inducing oxidative stress, which results in double-strand breaks and thus can lead to oncogenic and tumor suppressor mutations. This promotes tumor initiation and development. It has been shown that infection induces the expression of tumor protein 53 (Tp53) and Proliferating Cell Nuclear Antigen (PCNA) probably as a consequence of the damage generated. Also, the parasite could influence telomere attrition and its repair mechanisms, inducing apoptosis or senescence. However, it has also been observed that infection leads to an increase in antioxidant molecules that reduce DNA damage.

Infection amplifies the expression of Proliferating Cell Nuclear Antigen (PCNA), a protein implicated in DNA damage repair and prevention (Hassan et al., 2006; Wang, 2014). Consequently, it is reasonable to speculate that the parasite could serve as a tumor initiator. However, Escalante et al. (2006) discovered that infection with *T. cruzi* in rats led to an increased number of colonic crypts overexpressing metallothioneins, proteins capable of safeguarding against colorectal carcinogenesis by acting as antioxidants and averting tissue damage. Thus, it can be inferred that the parasite might positively influence tumor progression by acting as a genotoxic agent and negatively by protecting cells against DNA damage (Figure 5).

#### 1.4.3 Enable replicative immortality and evade growth suppressors

Cellular immortalization centers on the ability to sustain telomeric DNA to prevent senescence or apoptosis. Inflammation, exposure to infectious agents, and various oxidative stresses attack telomers and disrupt their repair mechanisms (Ilmonen et al., 2008). In this context, *T. cruzi* could impact this hallmark of cancer (Figure 5).

Interestingly, the absence of genomic integrity surveillance results in cells surviving even in the presence of telomere erosion, while eluding pathways that negatively regulate cell proliferation. This phenomenon hinges on the actions of tumor suppressor genes, such as the p53 tumor protein (Tp53), which, in response to signals indicating severe DNA damage, can trigger apoptosis and negatively regulate cell proliferation (Marei et al., 2021). In this context, *T. cruzi* infection induces Tp53 expression, but this is likely a consequence of the damage incurred by infected cells. A reduction in other tumor suppressors, such as caveolin-1, has also been reported (Mukherjee et al., 2004; Bouzahzah et al., 2006; Hassan et al., 2006). Consequently, analyzing how *T. cruzi* could influence these hallmarks is challenging, but changes in these signaling pathways are presumably induced post-infection. It is plausible that inhibiting host cell division fosters a more favorable environment for parasite replication while potentially inducing host cell proliferation as a mechanism to secure host cells for replication (Costales et al., 2009; Duran-Rehbein et al., 2017).

#### 1.4.4 Cellular senescence

Despite the potential advantages of senescence induction for tumor suppression to prevent additional mutagenic effects, senescent cells may stimulate malignant progression in specific contexts (Hanahan, 2022). Senescence entails a significant reduction in the ability of immune cells to eliminate pathogens, which leads to chronic infections. Conversely, microorganisms can provoke tissue stress, culminating in molecular and physiological changes in host cells that promote senescence (Scovino et al., 2021). Consequently, *T. cruzi* could influence this hallmark.

Indeed, chronic immune activation, uncontrolled and accelerated immunosenescence driven by parasite infection, results in host cell senescence (Albareda et al., 2009; Aguilera et al., 2018). For example, the infection triggers a rapid cellular stress response marked by DNA damage, followed by the induction of a senescence-like phenotype in infected NIH-3T3 fibroblasts, with senescent cells serving as parasite reservoirs (Guimarães-Pinto et al., 2018). Hence, *T. cruzi* may induce senescence in tumor cells, though whether this is beneficial or detrimental to tumor growth remains unclear (Figure 5).

#### 1.4.5 Resisting cell death

**FIGURE 6:**
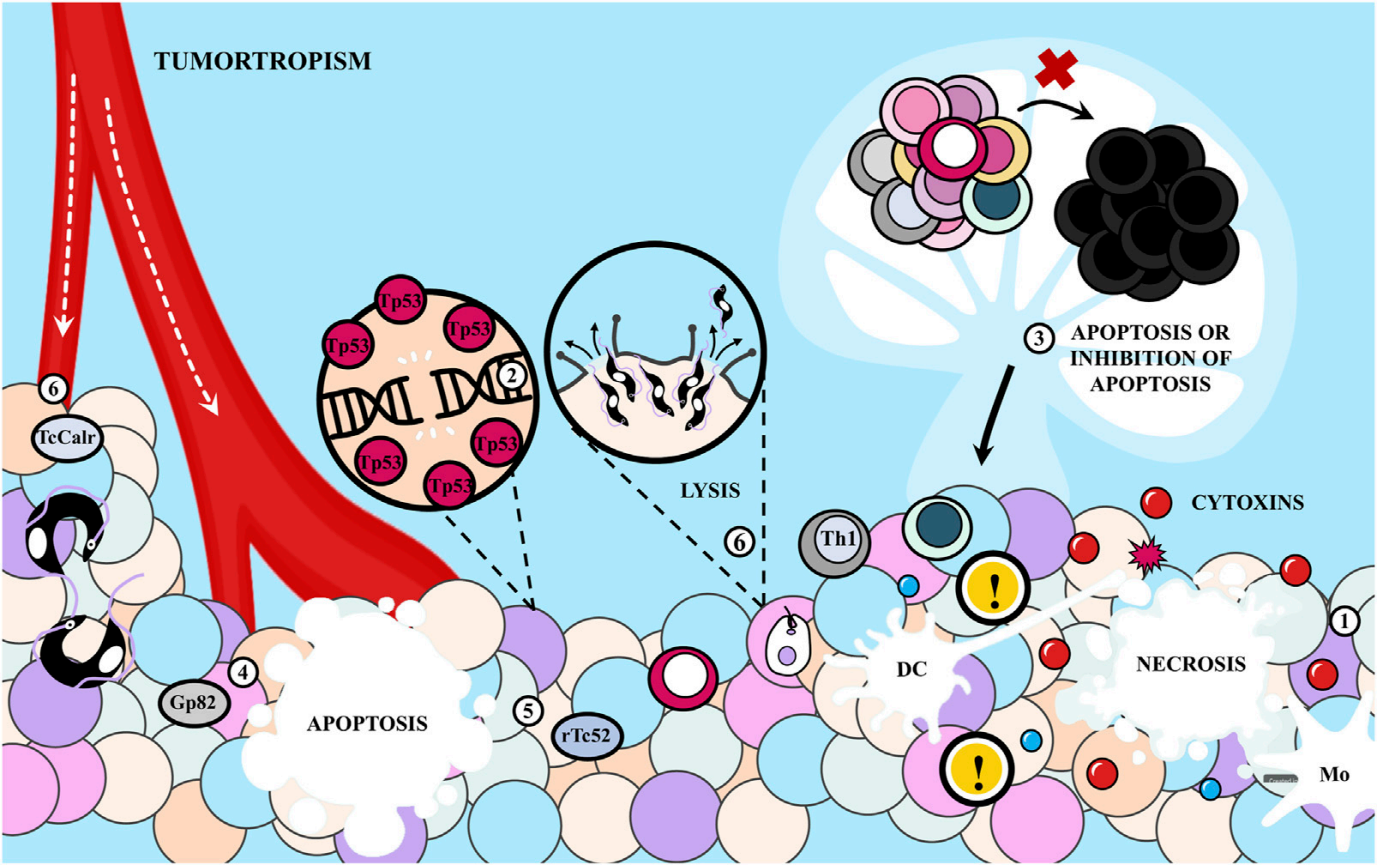
Cell death and *Trypanosoma cruzi*. *T. cruzi* can interfere with different types of cell death. (1) The parasite could produce cytotoxins that cause tumor necrosis, causing tolerogenicity or starting the anti-tumor immune response. The process of apoptosis induced by the parasite is also controversial. (2) The infection induces tp53 expression and there is evidence that (3) the infection promotes and inhibits immune cell apoptosis. (4) The *T. cruzi* Gp82 protein is able to specifically induce apoptosis of malignant cells while (5) the recombinant version of Tc52 (rTc52) protein induces apoptosis in splenic cells and human tumor cell lines and the *T. cruzi* Calreticulin (TcCalr) protein would cause immunological cell death. In addition, (6) there can be invasion and consequent lysis of neoplastic cells due to the tumortropism of the parasite, and this would lead to the release of tumor antigens that could initiate an antitumor response.

Cell death acts as a natural barrier preventing the survival and dissemination of malignant cells. However, neoplastic cells employ various strategies to evade this barrier, contributing not only to cancer initiation but also to the development of therapeutic resistance, recurrence, and metastasis (Hanahan and Weinberg, 2011). Cell death processes can be categorized as accidental, biologically uncontrolled (manifesting as lytic or necrotic death), or programmed (genetically directed, encompassing apoptosis and forms of necrotic cell death). Additionally, they can be classified as immunogenic or non-immunogenic, depending on neoplastic cell antigenicity, inflammation, and adjuvancy (Tan et al., 2021). *T*. *cruzi* can interfere with various types of cell death processes (Figure 6). This is of particular interest because it has been observed that tumor cells undergoing death induced by available therapies can mimic, at least partially, the behavior of pathogen-infected cells (Garg and Agostinis, 2017).

##### 1.4.5.1 Necrosis

It has been postulated that *T. cruzi* produces molecules with toxic properties affecting cancer cells (Roskin and Romanova, 1935; 1938b). This hypothesis finds support in experiments demonstrating the cytotoxic effects of parasite lysates on tumor cell cultures and in observations of antitumor effects in animal models that cannot be explained by alternative mechanisms (Melnikov et al., 2004; Zenina et al., 2008).

In *in vivo* experiments, Melnikov et al., 2004 investigated the antineoplastic effects of *T. cruzi* infection in L5178Y lymphoma and noted more extensive necrotic areas at various locations within the tumors of infected animals compared to non-infected animals. Notably, in the former, the necrotic areas were more prominent at the tumor periphery, distant from actively proliferating cells at the center. Since parasites were not detected within the tumor, the presence of parasitic molecules with cytotoxic potential modulating tumor growth was suggested (Melnikov et al., 2004).

Morillo et al. (2014) reported that infection, whether acute or chronic, reduced tumor development and increased the survival of mice challenged with a melanoma cell line. However, histopathological examinations revealed that areas of necrosis were associated with intracellular amastigotes found within parasitophorous vacuoles. Additionally, melanoma cells obtained from acutely infected mice exhibited reduced proliferative capacity when propagated in healthy mice, resulting in reduced tumor growth and prolonged survival. This phenomenon could be attributed to necrosis or apoptosis, as these cells, when inoculated, were likely phagocytosed by antigen-presenting cells, triggering a more effective antitumor immune response (Morillo et al., 2014).

In conclusion, whether *T. cruzi* metabolites exert a cytotoxic or cytostatic effect and inhibit tumor growth remains unclear (Melnikov et al., 2004). The high survival of both normal and cancer cells in extensively infected cultures, except for those invaded and lysed by the protozoan’s life cycle, provides evidence against the existence of a cytotoxin. While it is widely accepted that neither *T. cruzi* trypanomastigote nor amastigote forms produce toxins, it remains a hypothesis worthy of further investigation (Cabral, 2000). Furthermore, even within the context of necrosis induced by infection, the induction of tolerogenicity should be considered, as necrosis is not always immunogenic despite its inflammatory phenotype (Garg and Agostinis, 2017).

##### 1.4.5.2 Apoptosis

The induction of host cell apoptosis by *T. cruzi* remains a topic of debate. Apoptosis can be described as a deliberate, active process involving the sacrifice of specific cells for the greater benefit of the organism (Xu et al., 2019). In response to this, tumor cells have developed strategies to evade apoptosis, including the loss of function of the Tp53 tumor suppressor (Hanahan and Weinberg, 2011). Interestingly, *T. cruzi* infection induces Tp53 expression (Bouzahzah et al., 2006).

The presence of GPI anchors in the parasite membrane mediates the induction of apoptosis in infected macrophages (Freire-de-Lima et al., 1998). *T. cruzi* infection can also trigger apoptosis in placental tissue, specifically in human chorionic villi (Duaso et al., 2011). Nevertheless, immunosuppression observed during the acute phase of infection is thought to result from apoptosis of TL and BL in the thymus and secondary lymphoid organs (SLOs) (Borges et al., 2005; Ouaissi and Ouaissi, 2005; Mucci et al., 2006). However, it has been noted that infection inhibits early-stage death receptor-mediated apoptosis and that the parasite protein *trans*-sialidase (TS) can prevent neuronal cell apoptosis and rescue splenic TL from this form of cell death (Hashimoto et al., 2005; Mucci et al., 2006).

There is an apparent contradiction regarding cardiomyocytes. While some argue that chronic chagasic cardiomyopathy results from apoptosis of these cells, others have observed that invasion by the parasite suppresses this process (Aoki et al., 2004; Petersen et al., 2006; Morillo et al., 2014).

*T. cruzi* induces the expression of apoptotic regulators, which likely benefits the parasite by preventing host cell death before the completion of its intracellular replicative cycle (Costales et al., 2009; Chain et al., 2020).

Conversely, the recombinant form (J18) of the parasite’s Gp82 protein, expressed only in the metacyclic trypomastigote form and involved in invasion by disassembling the actin cytoskeleton, specifically induces apoptosis of Tm5 melanoma cells *in vitro*. This leads to the inhibition of tumor growth and an increase in *in vivo* survival rates (Cortez et al., 2006; Atayde et al., 2008).

Another aspect of this topic is Tc52, a protein released by *T. cruzi* with immunoregulatory activity. Its genetic fusion with a transporter protein induces apoptosis in splenic cells of BALB/c or CBA mice in a time- and dose-dependent manner. It also induces apoptosis in human tumor cell lines but not in normal cells like HeLa cells. The native Tc52 protein does not have this effect, suggesting that conformational changes in the recombinant protein result in apoptosis-inducing properties (Borges et al., 2005).

In summary, the extent of apoptosis induced during infection varies depending on the cell type and parasite strain under consideration. However, infection could induce apoptosis in neoplastic and immune cells, potentially altering the course of tumor development (Morillo et al., 2014).

##### 1.4.5.3 Lytic cell death: the tumortropism hypothesis

As previously mentioned, one of the defining characteristics of *T. cruzi* is its preference for specific tissues or organ systems (Cabral, 2000). Although the protozoan is capable of infecting and proliferating in almost all cell types *in vitro*, and parasites can be found in nearly all tissues, major foci are restricted to specific regions (Dvorak and Howe, 1976). For instance, Kallinikova et al. infected mice with a cardiomyotropic strain and found that the heart contained between 40% and 65% of the total parasites (Kallinikova et al., 2001). It has also been suggested that differential susceptibility to infection among different cell lines may depend on the presence of sialic acid residues on the cell surface (Vargas-Zambrano et al., 2013).

The concept of the anticancer activity of *T. cruzi* being based on tumortropism, the ability to preferentially infect and proliferate within host neoplastic cells compared to normal cells *in vivo*, has been proposed (Melnikov et al., 2004). The presence of amastigotes within cancer cells has been observed, and changes in the degree of tumortropism during the parasite’s life cycle positively correlate with its ability to induce an active anticancer response (Kallinikova, 1964; Kallinikova and Kats, 1968; Kallinikova et al., 2001).

Kallinikova’s group observed that in mice challenged with sarcoma-80 and infected with either virulent or avirulent *T. cruzi* strains, there was a delay, stabilization, or regression of tumor growth. Furthermore, the presence of the tumor led to the redistribution of parasites among tissues, with the tumor being one of the most invaded tissues. In co-culture experiments, a more successful infection of malignant cells compared to renal cells and fibroblasts was observed. The research showed that one strain of *T. cruzi* made no distinction between cardiomyocytes and cancer cells (Kallinikova et al., 2001).

The invasion and subsequent lysis of neoplastic cells that could occur during the typical course of *T. cruzi* infection may release TAgs, initiating an anticancer response. This may provide DCs with Ags and inflammatory stimuli, leading to the activation of CTL through antigen cross-presentation (Chen and Mellman, 2013; MacNabb et al., 2022).

However, an antitumor effect has been observed even when cancer cells were not parasitized, and the infection was only minimally present in the tumor stroma (Hauschka and Goodwin, 1948).

In addition, the tumortropic properties of *T. cruzi* are less pronounced *in vivo* than in co-cultures (Kallinikova et al., 2001). May be that not all *T. cruzi* strains have tumortropism and probably the type of tumor is relevant here (Melnikov et al., 2004). In any case, the antitumor effect observed in mice with CD in the chronic stage or after immunization with parasite extracts cannot be attributed to this mechanism (Morillo et al., 2014; Ubillos et al., 2016; Freire et al., 2022).

In some models, tumor growth resumes its typical rate when infection is treated (Hauschka and Goodwin, 1948; Ramírez-Toloza et al., 2015). Additionally, the anticancer effects become more significant with elevated parasitemia or the use of more virulent strains, emphasizing the importance of parasite presence for this mechanism (or the toxin hypothesis). However, the dependence on parasitemia does not appear to be crucial, as a considerable anticancer effect has been observed even at low parasitemia (Kallinikova et al., 2001).

##### 1.4.5.4 *T. cruzi* Calreticulin and immune cell death

Human tumor tissues exhibit significantly higher levels of Calreticulin (HuCalr) compared to normal tissues. This is associated with a favorable prognosis as it triggers the activation of an adaptive immune response (Ramírez-Toloza et al., 2016). Therefore, *T. cruzi* Calreticulin (TcCalr) secreted by the parasite may also be significant.

TcCalr possesses remarkable adjuvant potential, superior to that of HuCalr, and seems to be effective across various tumor types (Abello-Cáceres et al., 2016; Cruz et al., 2020). TcCalr facilitates the presentation of peptide sequences in the Major Histocompatibility Complex (MHC) that are distinct from those of tumor cells due to structural and sequential differences. This increases the immunogenicity of neoplastic cells (Cruz et al., 2020).

The stress condition induced by TcCalr leads tumor cells to externalize their Calreticulin (Calr), serving as an “eat-me signal.” This triggers the recruitment of the complement system and increases the phagocytosis of tumor cells by DCs. In SLOs, antigenic peptides derived from TcCalr, acting as TAg and other Ags, are presented. This activation of CTL through antigen (Ag) cross-presentation allows them to infiltrate tumors and metastatic sites, eliminating cancer cells. Activation of HTL through MHC II presentation, with subsequent stimulation of BL and ADCC against tumor cells, is also a potential mechanism to consider (Ramírez-Toloza et al., 2016).

Also, the TcCalr (recombinant (rTcCalr) or native) binds to tumor cells and can provide strong signals revealing the presence of the tumor to the tolerogenic immune system. In co-cultures of tumor cells with Ag-presenting cells that were treated with rTcCalr, neoplastic cells were engulfed and matured DCs and were able to activate and expand TL efficiently (Cruz et al., 2020). Furthermore, subcutaneous peritumoral inoculation of rTcCalr *in vivo* has shown to increase local TL infiltration and slow tumor development of a mammary adenocarcinoma (TA3 cell line) by increasing phagocytosis and modulating the expression of membrane molecules that correlate with improved tumor immunogenicity (Sosoniuk-Roche et al., 2020). Indeed, the combined treatment of Survivin -a tumor-associated Ag- and rTcCalr inhibited tumor growth of a melanoma cell line expressing Survivin and this was associated with the tumor phagocytosis (Aguilar-Guzmán et al., 2014).

#### 1.4.6 Altered neuronal signaling

Nerve and neuronal signaling are vital for tumor growth and survival, actively modulating tumor microenvironment and providing proliferative signals to neoplastic cells (Wang et al., 2020; Zahalka and Frenette, 2020). Consequently, the density of nerve fibers in tumor tissue correlates with cancer aggressiveness.

Myenteric neurons are recognized as a key factor in the development of early cancerous lesions in the colon. Experimental ablation of myenteric neurons in rats has been shown to protect against neoplasms in the colon. The development of megacolon during the chronic phase of *T. cruzi* infection is associated with the loss of innervation (Kannen et al., 2015). Additionally, Garcia et al. (1996) observed that the number of distal colon carcinomas in rats with experimental megacolon (without infection) was lower than in the absence of megacolon. Hence, a potential relationship between megacolon and oncoprotection at the colon level likely exists, perhaps due to the imbalance of neuroendocrine mediators in the denervated colon influencing tumor cell metabolism, survival, and proliferation (Novaes et al., 2016).

#### 1.4.7 Sustaining of proliferative signaling

Cell proliferation is an essential process for cancer growth. Neoplastic cells primarily exhibit chronic proliferation by disrupting signaling pathways governing cell cycle progression (MacCarthy-Morrogh and Martin, 2020). As previously discussed, *T. cruzi* infection induces alterations in these signaling pathways. Transcriptomic changes affecting cell cycle regulators have been documented in various cell types under different experimental conditions following *T. cruzi* infection. Nevertheless, these findings exhibit limited consistency. In the case of infected fibroblasts, Costales et al. (2009) and Li et al. (2016b) observed an initial enrichment in functions related to cell cycle progression, mitosis, and cell division. However, this trend reversed 24 h post-infection, with a rapid decline in the expression of host cell cycle genes, ultimately impeding host cell cycle progression. Furthermore, observations of altered cytokinesis in infected cells have been reported.

**FIGURE 7:**
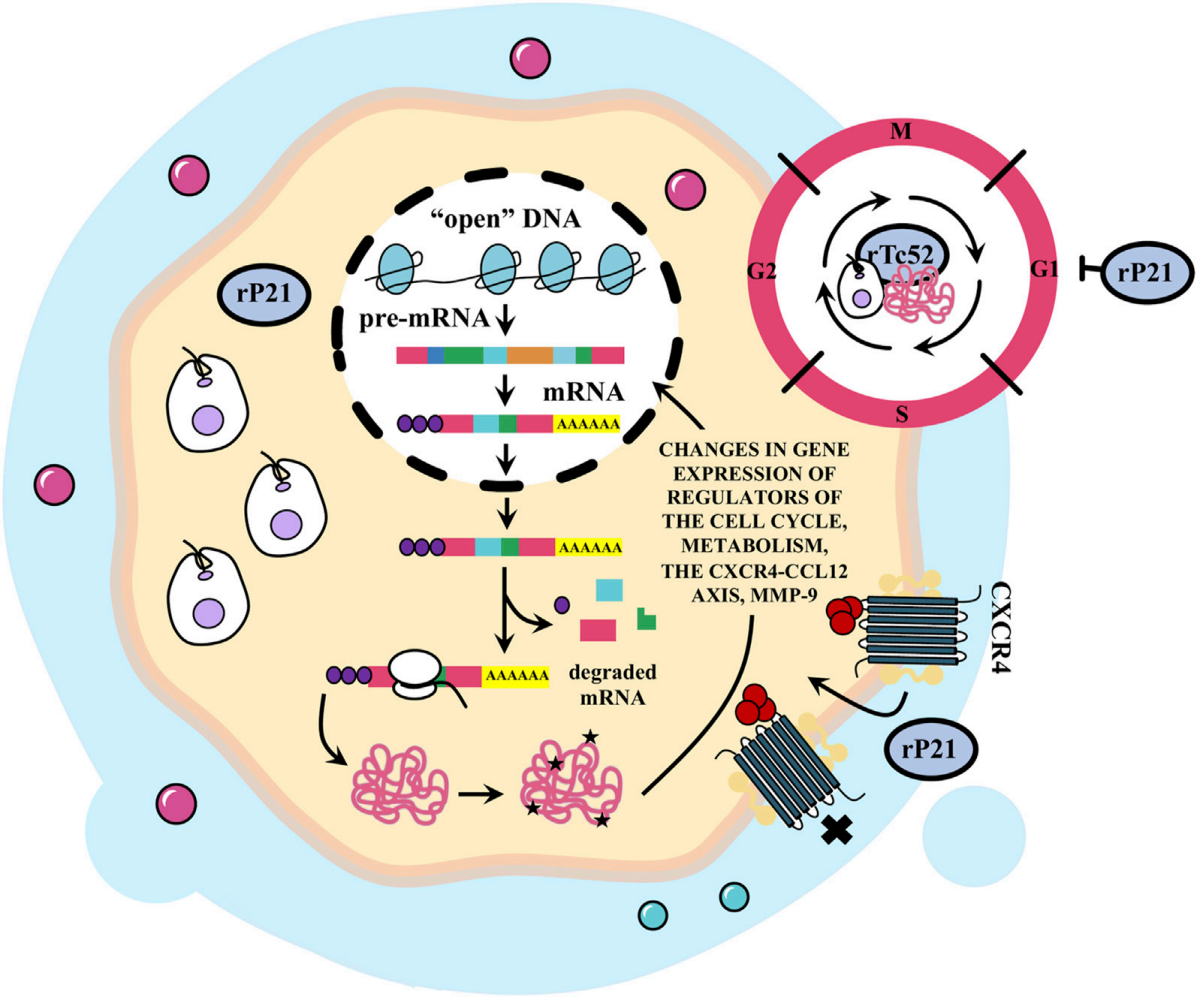
*Trypanosoma cruzi* produces changes in the cell cycle and affects tumor cell metabolism and invasiveness. Infection with *T. cruzi* could affect cancer development through changes in gene expression of cell cycle and metabolic regulators. It has been reported that treatment with recombinant P21 (rP21) stops the cell cycle of neoplastic MDA-MB-231 cells in the G1 phase, although infection can as well promote the proliferation of this cell line (discussed in the text). This protein also decreases the expression of CXCR4 and the migration marker mmp-9 gene in tumor cells inducing the internalization, desensitization or blockage of CXCR4. This inhibits the formation of metastatic foci. The recombinant Tc52 (rTc52) protein released by *T. cruzi* induces apoptosis in splenic cells of mice and in human tumor cell lines but not in normal cells. However, it has been reported that the intracellular production of Tc52 from *T. cruzi* stimulates the growth of macrophages and fibroblasts.

Notably, exposure to conditioned medium from a melanoma culture infected with *T. cruzi* led to a complete inhibition of tumor cell proliferation (Melnikov et al., 2004). In addition, *T. cruzi* infection rendered TL cells unresponsive to mitogenic stimuli (Borges et al., 2005). Similarly, treatment with recombinant P21 (rP21) arrested the cell cycle of the neoplastic cell line MDA-MB-231 in the G1 phase but had no such effect on the non-tumor cell line MCF-10A (Borges et al., 2020). On the contrary, conflicting data suggest that *T. cruzi* infection may promote proliferation in tumor cells (Azevedo Silveira et al., 2023). Moreover, *T. cruzi* infection has been shown to induce proliferation in a trophoblastic cell line and in hepatic epithelial cells through the activation of the ERK1/2 MAPK pathway (Bouzahzah et al., 2006; Droguett et al., 2017). Additionally, the infection increased the expression of cyclin D1 and/or PCNA, critical regulators of proliferation, in cultures of smooth muscle cells and myocardial tissue in infected mice (Hassan et al., 2006). Exposure of a human astrocytoma cell line to an Ag from *T. cruzi* trypomastigotes resulted in increased cell proliferation, with the number of tumor cells directly correlating with Ag concentration (Duran-Rehbein et al., 2017). Furthermore, intracellular production of Tc52 from *T. cruzi* stimulated macrophage and fibroblast growth (Ouaissi and Ouaissi, 2005). These observations collectively suggest that *T. cruzi* may either induce or inhibit cell proliferation by interfering with the underlying regulatory pathways (see Figure 7).

#### 1.4.8 Reprogramming energy metabolism

Cancer cells undergo metabolic reprogramming to sustain their uncontrolled growth and proliferation (Hanahan and Weinberg, 2011). It is well-established that *T. cruzi* significantly modulates host cell metabolism. In various cell types, changes in the abundance of cytokine-independent transcripts resulting from infection have been observed. These changes are closely associated with the metabolic consequences of intracellular parasitism, with induction of metabolic pathways and inhibition of degradative pathways (Melnikov et al., 2004; Costales et al., 2009; González et al., 2019).

Evidence suggests that *T. cruzi* competes with tumor cells for essential nutrients. The infection in mice leads to generalized host exhaustion and weight loss (Hauschka and Goodwin, 1948). Based on this evidence, it is plausible that alterations in the metabolic profile of neoplastic host cells could either promote or inhibit tumor growth (see Figure 7).

#### 1.4.9 Activation of invasion and metastasis

Metastasis, characterized by the spread of abnormal cells beyond their normal confines and their invasion of adjacent regions or distant organs, is the primary cause of death in cancer (Steeg, 2016). Research by Melnikov et al. (2004) demonstrated a reduction in liver metastases from L5178Y-R lymphoma during the acute phase of *T. cruzi* infection, despite the absence of parasites in the liver. In a separate study, Azevedo Silveira et al. (2023) reported that *T. cruzi* inhibited the migration capacity of MDA-MB-231 tumor cells.

Chemokines and their receptors play a crucial role in regulating the migration and metastasis of neoplastic cells. The CXCR4/CXCL12 axis is instrumental in controlling migration and survival under suboptimal conditions (Balkwill, 2004). Notably, tumor cells express high levels of CXCR4 and produce CXCL12, which acts in an autocrine and paracrine manner (Liekens et al., 2011). rP21 was observed to decrease the expression of CXCR4 and the MMP-9 gene, a marker of migration, selectively in tumor cells. This evidence suggests that rP21 may induce internalization, desensitization, or blockade of CXCR4, thereby reducing CXCL12-induced chemotaxis and invasion (Borges et al., 2020). Therefore, this mechanism may contribute to the inhibition of metastatic focus formation (see Figure 7).

#### 1.4.10 Aberrant glycosylation

Aberrant glycosylation, characterized by deviations from normal glycosylation processes, is often associated with inflammation and cancer. Emerging evidence underscores the pivotal role of glycosylation in various stages of tumor progression (Munkley and Elliott, 2016). In neoplastic cells, *O*-glycans exhibit immature and/or truncated structures, due to reduced expression of specific glycosyltransferases, leading to the accumulation of altered glycan structures like Tn and sialyl-Tn Ags. *N*-glycans, conversely, manifest as long, branched, and hypersialylated structures (dos Reis et al., 2022). Both forms of aberrant glycosylation confer upon these structures the classification of tumor-associated carbohydrate Ags (Campo et al., 2014).

Notably, glycosylation is a prominent player in parasitic infection (Rodrigues et al., 2015). Mucins, which envelop and shield *T. cruzi*, contribute to the establishment of a persistent infection by subverting the host’s immune response against the parasite (Buscaglia et al., 2006). These mucins are heavily glycosylated at Thr, Ser, and Pro residues and are GPI-anchored (Acosta-Serrano et al., 2001). Notably, they feature a unique type of glycosylation comprising sialylated *O*-glycans attached to the protein backbone via α-GalNAc residues, a process catalyzed by TS in the presence of sialic acid donors (Pereira-Chioccola et al., 2000).

*T. cruzi* mucins exhibit substantial differences in glycosylation patterns compared to those in mammals and share greater similarities with mucin-like molecules implicated in lymphocyte trafficking than with epithelial mucins (Buscaglia et al., 2006). Additionally, *T. cruzi* expresses TAg Tn and sialyl-Tn that can provoke an effective immune response against cancer cells (Freire et al., 2003). There is a possibility that a TS variant capable of transferring sialic acid to α-GalNAc residues exists, given that this enzyme is functionally related to host sialidases. TS exhibits variable specificities for sialic acid donors, allowing it to play roles in a diverse array of host systems (Campo et al., 2014). It is conceivable that *T. cruzi* TS may alter the glycosylation pattern of neoplastic cells. This action is believed to involve the removal of sialic acid from cell membranes, potentially leading to apoptosis (Higuchi, 2004).

**FIGURE 8:**
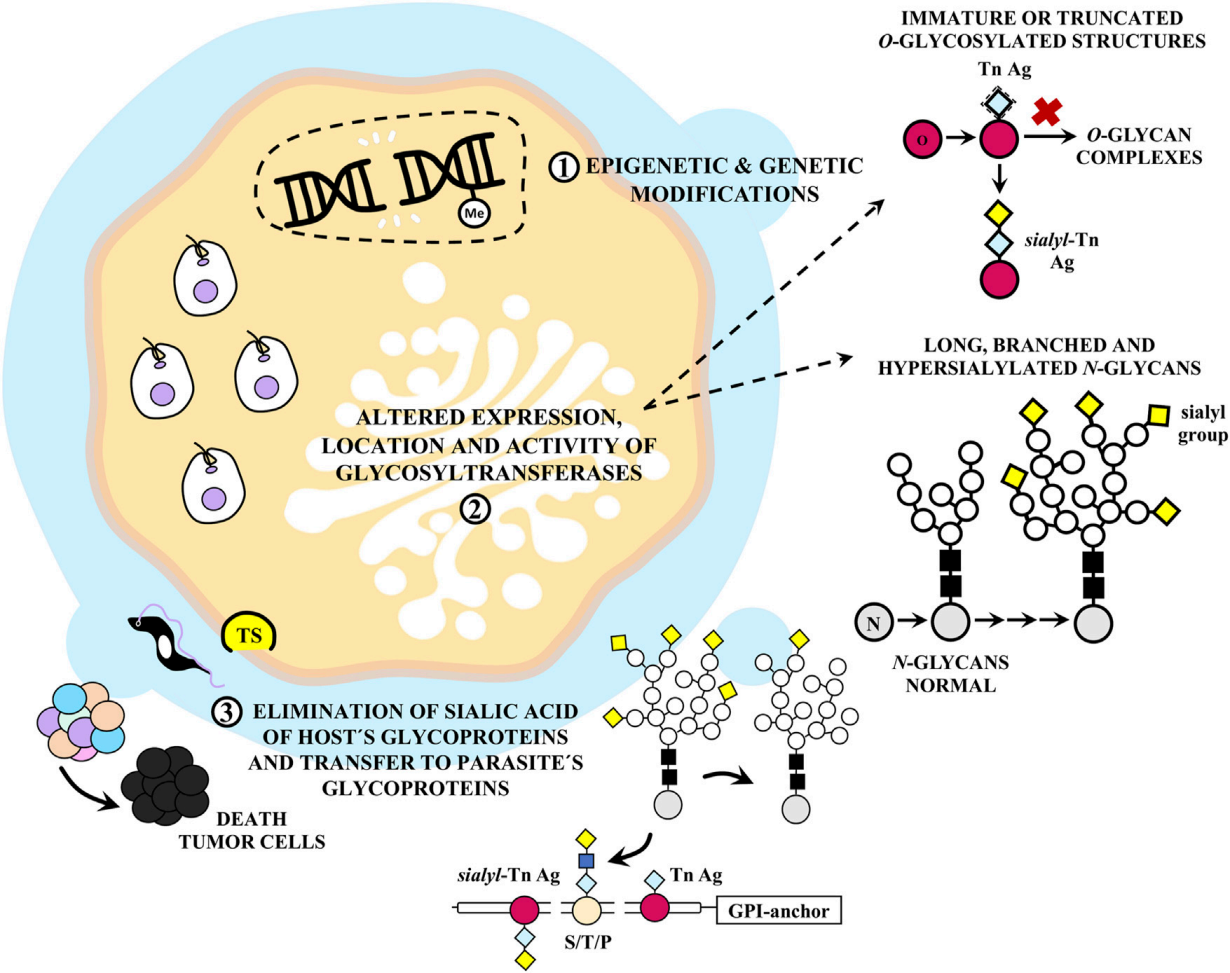
Aberrant glycosylation of tumor cells by *Trypanosoma cruzi* infection. (1) *T. cruzi* can act as carcinogen and damage the host cell DNA. (2) This can affect the expression, activity and localization of glycosyltransferases. In neoplastic cells, *O*-glycans are characterized by immature and/or truncated structures such as Tn and sialyl-Tn antigens (Ags), whereas *N*-glycans are usually long, branched and hypersialylated. The mucins that coat and protect *T. cruzi* are glycosylphosphatidylinositol (GPI) anchored and highly glycosylated at Thr (T), Ser (S), and Pro (P) residues. They contain a unique type of glycosylation pattern consisting of several sialylated *O*-glycans attached to the protein backbone via α-GalNAc residues by the action of the enzyme *trans*-sialidase (TS), which catalyzes this binding in the presence of host sialic acid donors. These mucins show notable differences from those found in mammals, although *T. cruzi* also expresses the tumor Ags Tn and sialyl-Tn. It is proposed that (3) TS could act on neoplastic cells causing changes in the glycosylation pattern, eliminating sialic acid from cell membranes and thus increasing their apoptosis.

Even in small quantities, TS can induce apoptosis in thymus and organized lymphoid structures by generating an incorrect sialylation pattern in immune cells (Mucci et al., 2002). Although the protein is distributed systemically early during infection, primarily during the acute phase when immune system damage is most apparent, the circulating enzyme level is subsequently controlled by neutralizing antibodies. TS is expected to exert its most significant effects during the acute infection phase. Local *in situ* concentrations resulting from infected cells may be adequate to induce apoptosis in neighboring cells (Leguizamón et al., 1999). These mechanisms are summarized in Figure 8.

#### 1.4.11 Inducing vasculature

Solid tumors are typically highly vascularized, rendering them susceptible to insufficient blood supply. Consequently, anti-angiogenesis is a preferred strategy due to the low likelihood of encountering resistance, owing to the low mutagenic potential of endothelial cells. Anti-angiogenesis not only curbs tumor growth but also inhibits metastasis by limiting the expansion of neoplastic cells and preventing their dissemination through aberrant blood vessels (Ramírez et al., 2012).

Angiogenesis is a complex, multistep process driven by pro-angiogenic factors. To establish new blood vessels, endothelial cells must proliferate, migrate through the extracellular matrix surrounding tumor tissue to facilitate nutrient and oxygen supply, and establish a medium for waste removal (Folkman, 2005). The “angiogenic switch” is initiated by the TM and drives endothelial cell multiplication while avoiding apoptosis (Ramírez et al., 2012). Anti-angiogenic agents primarily target actively growing neoplastic tissues, as they are primarily effective on emerging blood vessels (Molina et al., 2005). This observation may elucidate the association between the time lapse between *T. cruzi i*nfection and tumor inoculation and the observed oncoprotective effect in certain models.

This protective mechanism may represent an evolutionary adaptation benefiting the parasite by increasing host survival and enabling the parasite to expand its genome. Decreasing angiogenesis may also impede immunocompetent cell access to parasite sites and subsequently dampen the inflammatory response, which serves the aggressor’s interests, although reduced inflammation also benefits the host (Ramírez et al., 2012). The *T. cruzi* P21 protein exhibits anti-angiogenic properties. In its recombinant form, it elevates the production of sFlt-1 by macrophages, a soluble molecule that inhibits endothelial cell proliferation. It also impedes endothelial cell proliferation by binding to CXCR4 (Teixeira et al., 2017; 2019; Borges et al., 2020).

**FIGURE 9:**
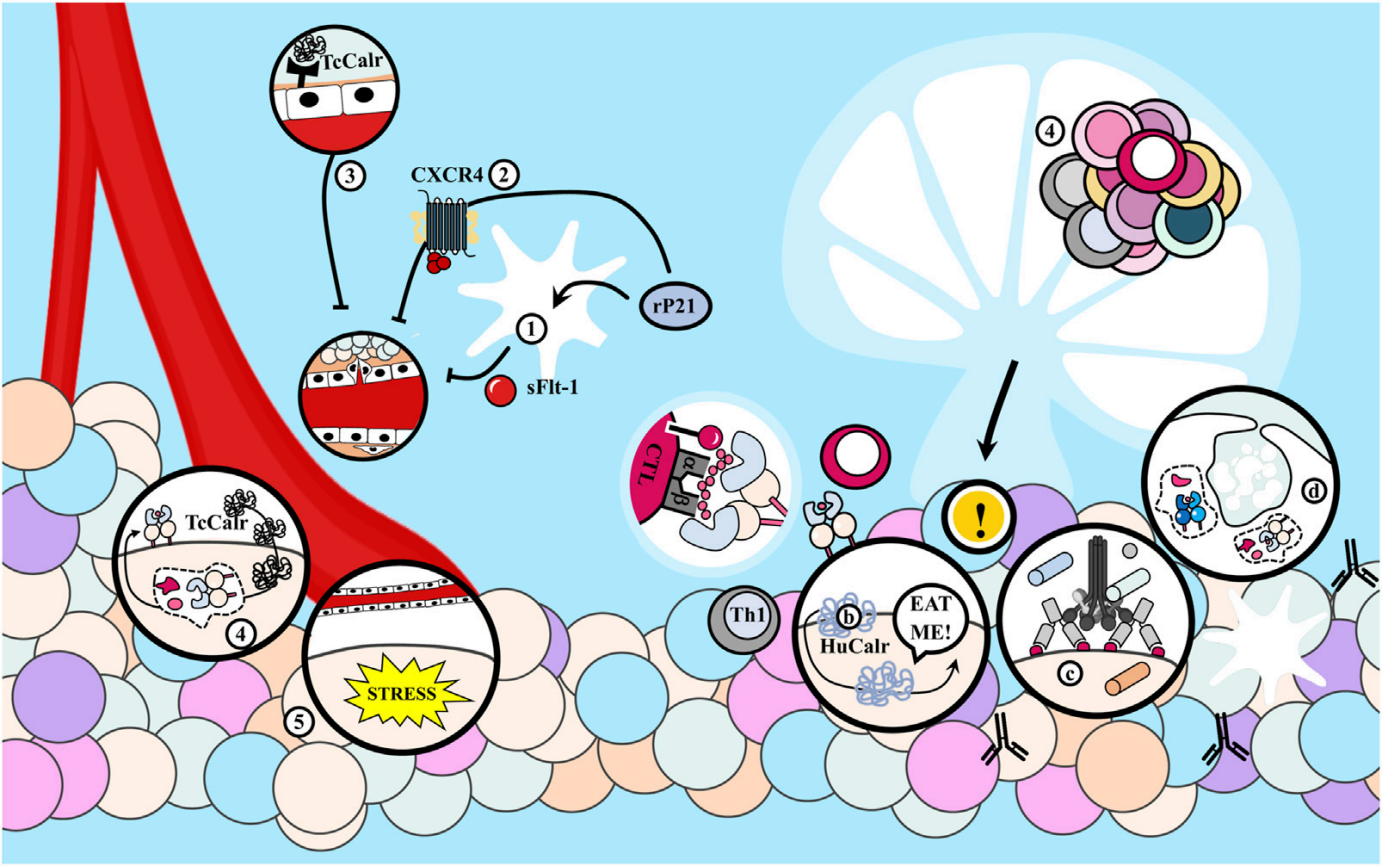
*Trypanosoma cruzi* inhibits tumor angiogenesis. (1) The recombinant P21 (rP21) protein of the parasite increases the production of sFlt-1 by macrophages, which inhibits endothelial cell proliferation. (2) Also, by binding to CXCR4 in endothelial cells, this protein inhibits their proliferation. (3) *T. cruzi* Calreticulin (TcCalr) protein interacts directly with endothelial cells by binding to Scavenger receptors and inhibits their proliferation, migration and morphogenesis. (4) Also, TcCalr likely allow the presentation, in the context of Major Histocompatibility Complex (MHC), of peptide sequences different from those of tumor cells, increasing the immunogenicity of neoplastic cells. On the other hand, (5a) the stress condition generated by anti-angiogenic mechanisms of this protein causes tumor cells to externalize their Calreticulin (HuCalr). (5b-c) This acts as an “eat me” signal by recruiting the complement and (5d) increases phagocytosis of tumor cells. In turn this activates CD8^+^ T lymphocytes (CTL) by cross-presentation, which infiltrate the tumor and metastasis and eliminate cancer cells, and CD4^+^ T cells through MHC II presentation, with stimulation of B cells and the resulting antibody-dependent cellular cytotoxicity (ADCC) against tumor cells.

Similarly, as previously mentioned, TcCalr demonstrates anti-angiogenic characteristics (Ramírez-Toloza et al., 2020). Research indicates that TcCalr directly interacts with endothelial cells by binding to scavenger receptors, inhibiting their proliferation, migration, and morphogenesis, thus restraining tumor growth (Figure 9). Peritumoral administration of recombinant TcCalr in mammary adenocarcinoma and melanoma models leads to reduced tumor volume *in vivo*, with effects similar to those of parasitic infection. Importantly, the effects are reversible by anti-TcCalr antibodies (Toledo et al., 2010; Ramírez-Toloza et al., 2015; Abello-Cáceres et al., 2016). This indicates that TcCalr contributes significantly to the observed *in vivo* anti-tumor effects.

The antitumor influence of *T. cruzi* infection or immunization with epimastigote lysates on mammary tumor development appears to rely heavily on the N-terminal fragment of the protein (nTcCalr) (López et al., 2010; Peña Álvarez et al., 2020). Thus, given the existing evidence, the previous hypothesis of nutrient competition between tumors and parasites leading to tumor inhibition seems less likely (Ramírez-Toloza et al., 2015).

### 1.5 The relationship between Chagas disease and cancer in the clinical setting

#### 1.5.1 Gastrointestinal manifestations of Chagas disease and cancer

The chronic gastrointestinal manifestations of CD primarily result from the impairment of the enteric nervous system caused by *T. cruzi* infection. Patients may develop gastrointestinal tract dilatation and motor disorders, with megacolon being the most common manifestation, followed by megaesophagus, and a combination of megacolon and megaesophagus (Matsuda et al., 2009).

##### 1.5.1.1 Chagasic megaesophagus

Physiopathologically, chagasic megaesophagus shares similarities with idiopathic achalasia due to nerve cell destruction caused by direct parasitism, inflammatory, and/or autoimmune mechanisms (Zucoloto and de Rezende, 1990; Munari et al., 2019). Chagasic megaesophagus is associated with an increased risk of esophageal cancer (Manoel-Caetano et al., 2009). However, the frequency of these tumors in patients with chagasic megaesophagus varies significantly, with a 10 to 50-fold increased risk compared to the general population (Campanella et al., 2018). These differences may be due to variations in the study populations.

It is essential to note that the association between cancer and chagasic megaesophagus is not a direct one between cancer and *T. cruzi* infection or CD, as no increase in the frequency of esophageal cancer is observed in CD patients without megaorgan manifestations. *T. cruzi* infection alone is insufficient to increase the occurrence of esophageal cancer, and a similar trend is seen in patients with idiopathic achalasia (Gullo et al., 2012). One possible explanation for this phenomenon is the dilatation of the organ and resultant alimentary stasis, which causes chronic mucosal irritation and an increase in epithelial cell proliferation (Adad et al., 1999). This theory is partially supported by observations that esophageal tumors typically occur in the middle third segment, whereas in patients with megaesophagus, the most frequent tumor location is the distal third segment, where esophageal stasis is prominent (Martins et al., 2019).

Another explanation could be related to the transformation of dietary nitrates into nitrites mediated by bacteria in the organ’s lumen. The chronic contact of these carcinogens with the esophageal mucosa might play a role, especially considering that stasis leads to bacterial overgrowth (Pajecki et al., 2003).

Several studies have identified specific genetic alterations in esophageal carcinomas of chagasic megaesophagus patients, including mutations in tp53, pik3ca, fhit, cdkn2a, p16, mib1, microsatellite instability, and aneuploidies of chromosomes 7, 11, and 17 (Manoel-Caetano et al., 2009; Bellini et al., 2012; Lacerda et al., 2017; Campanella et al., 2018; Munari et al., 2018). This suggests that carcinogenesis in the context of chagasic megaesophagus may be influenced by host genetic factors, with the parasite-host interaction contributing to the chronic inflammation driving this process (van Tong et al., 2017).

There’s also a case report describing a patient with chagasic megaesophagus who developed esophageal leiomyosarcoma. This is attributed to the inflammatory process affecting other layers in addition to the frequent myositis, hypertrophy, and hyperplasia of muscle fibers. However, epithelial cells proliferate more than muscle cells, making the observed association incidental (Adad et al., 1999).

##### 1.5.1.2 Chagasic megacolon

Despite the presence of risk factors for colon cancer in CD-associated megacolon, such as chronic constipation and altered motility, which could prolong the contact of potential dietary and metabolic carcinogens with colonic cells, hyperplasia, mucosal ulcers, inflammation, and transient immunosuppression, a retrospective study involving 894 cases of megacolon found no preneoplastic lesions or colon cancer (Oliveira et al., 2001; Garcia et al., 2003). Thus, it seems that megacolon is associated with a lower frequency of colorectal cancer (Adad et al., 2002).

Colonic tumors in patients with chagasic megacolon are not typically found in the dilated segment of the megacolon, which is often the rectosigmoid part where most colorectal malignancies occur in the general population (Oliveira et al., 2001). Regarding the mechanism, it is known that the development of megacolon involves loss of muscle innervation (da Silveira et al., 2007). That is, during the megacolon there is a clear damage of myenteric neurons and a high proliferation related to hyperplasia occurs. However, the risk of colon cancer within the region of chagasic megacolon is reduced because myenteric neuronal density is altered. This supports the hypothesis that the enteric nervous system plays an important role in colon carcinogenesis (Kannen et al., 2015).

It is essential to consider that megacolon’s association with colon cancer is not only related to the nervous system but may also involve the immune system. There is an increased entry of CTL into enteric lymph nodes in megacolon patients, indicating a potential role of the immune system that requires further investigation (da Silveira et al., 2007).

#### 1.5.2 Chagas disease and cardiac neoplasms

Most symptomatic CD patients experience cardiac damage, including cardiomyopathy and heart rhythm abnormalities, due to the parasite’s infection of various cardiovascular cell types (Hassan et al., 2006; Matsuda et al., 2009). However, there appears to be no direct correlation between heart disease and cardiac neoplasms (Adad et al., 1999). Cardiac neoplasms do not seem to be frequent causes of death or show an increased frequency in patients requiring cardiac transplants due to these heart diseases (de Menezes et al., 1989; Da Consolação Vieira Moreira and Cunha-Melo, 2020).

#### 1.5.3 Chagas disease and gynecologic cancer

While there are no reports of gynecological clinical manifestations of CD, the association of this pathology with different tumors has led to the study of its relationship with gynecological neoplasms. The general consensus is that CD is neither a risk factor nor a protective factor for the development of gynecologic tumors (Dominical et al., 2010). However, a higher frequency of malignant gynecologic neoplasms has been observed in CD patients, which is likely attributable to the inflammatory process caused by *T. cruzi*. Also, a case-control study has reported that 27% of women who had a uterine leiomyoma -a benign tumor of the smooth muscle-were serologically positive for CD, compared with 16% of controls. In this case, it was proposed that parasitism of the uterine muscle fiber could increase the incidence of myomas, since parasites have been observed *in utero*. The same could occur as in chagasic megaesophagus, where myositis, hypertrophy or hyperplasia of muscle fibers were observed (Murta et al., 2002).

#### 1.5.4 Pharmacological intervention for Chagas disease and cancer

Only two drugs, benznidazole and nifurtimox, are licensed for the treatment of CD (Pérez-Molina and Molina, 2018). These drugs eliminate the parasite by inducing oxidative or reductive damage (Ribeiro et al., 2007).

There are no elements to suggest that the potential role of anti-trypanosome treatment in the development of cancer should be ignored, beyond the fact that it is a dosing schedule that does not exceed 90 days and is generally administered in the acute stage of the disease where efficacy is higher, although there is evidence that some benefits are observed if treatment is performed even when the disease is in its chronic stage (Pecoul et al., 2016; Pérez-Molina and Molina, 2018).

While benznidazole has been found to be mutagenic and associated with lymphomas in animal studies (Teixeira et al., 1990), others have observed that the compound caused a reduction in nitric oxide synthesis in treated infected mice; consequently, this contributed to protect against *T. cruzi*-induced oxidative DNA damage, minimizing parasite-induced genotoxicity (Ribeiro et al., 2007).

Li et al. (2016a) have found that benznidazole is a hypoxia-activated cytotoxin that can specifically kill clonogenic tumor cells that are under severe hypoxic conditions or tumor-initiating cancer cells It probably acts by inducing double-strand breaks, just as on *T. cruzi* DNA; the same has also been reported for nifurtimox (Li et al., 2017). In addition, some clinical observations do not indicate modifications in the incidence of neoplasms in CD patients treated with benznidazole (Andrade et al., 2003).

In contrast, nifurtimox exerts an inhibitory effect on the proliferation and/or viability of neuroblastoma cell lines, while sparing normal cells. This effect is attributed to the induction of apoptosis through the generation of reactive oxygen species, which subsequently inflict damage upon biologically significant biomolecules, resulting in cell death. This process is accompanied by a notable reduction in lactate production, attributable to diminished lactate dehydrogenase enzyme activity and downregulation of the N-Myc protooncogene (Saulnier Sholler et al., 2006; 2009; Cabanillas Stanchi et al., 2015). Remarkably, a case report documents a neuroblastoma patient with concomitant CD, who, upon treatment with nifurtimox, exhibited a substantial and unforeseen reduction in tumor size. Notably, this neoplasm had previously exhibited resistance to conventional cancer chemotherapy regimens (Saulnier Sholler et al., 2006).

#### 1.5.5 Cancer and reactivation of Chagas disease

Bocchi et al. (1998) conducted a study involving 16 patients diagnosed with CD who underwent cardiac transplantation, subsequently experiencing disease reactivation. Their investigation revealed that six of these patients developed neoplasms, comprising three cases of lymphoproliferative disorders, two cases of Kaposi’s sarcoma, and one case of squamous cell carcinoma. This phenomenon has been attributed to the immunosuppressive pharmacological regimen administered to prevent organ rejection. Nevertheless, it is worth noting that the role of disease reactivation and the potential pro-tumorigenic effects of the parasite, in conjunction with anti-trypanosome treatment as recommended in such cases, should not be dismissed.

Indeed, one study documented cases of CD reactivation in patients with hematologic malignancies, encompassing acute lymphoblastic leukemia, acute lymphocytic leukemia, Hodgkin’s lymphoma, non-Hodgkin’s lymphoma, and follicular lymphoma (d’Avila Rosenthal et al., 2016). These instances were also attributed to aggressive immunosuppressive treatment, in light of reports of reactivation following organ transplantation and in the context of autoimmune diseases necessitating pharmacological immunosuppression and HIV-AIDS immunocompromise. Importantly, such reactivation did not manifest in patients afflicted with other tumors, who likewise underwent comparable treatments, albeit with a lower risk of infections as reported (Ferreira et al., 1997; Gallerano et al., 2007; Burgos et al., 2012; Campo et al., 2014; Castillo et al., 2014; Kransdorf et al., 2014; Lattes and Lasala, 2014; Gattoni et al., 2015; d’Avila Rosenthal et al., 2016; Vacas et al., 2017; Da Consolação Vieira Moreira and Cunha-Melo, 2020; Czech et al., 2021; Ringer et al., 2021).

Moreover, reactivation has exclusively been observed in cases of hematological malignancies (Kohl et al., 1982; Metze et al., 1991; Rezende et al., 2006; Altclas et al., 2014; Jimenez-Marco et al., 2014; Garzón et al., 2015). Regrettably, the impact of disease reactivation on tumor prognosis remains unexplored, which could provide insight into whether heightened parasitemia exerts any influence. Specifically, it remains uncertain whether this effect is purely opportunistic or if it plays a substantial role, especially when considering that chronically infected Swiss mice exposed to diverse *T. cruzi* strains and subjected to immunosuppressive treatment did not exhibit neoplastic proliferation in their lymphoid tissues. This observation holds significance given clinical reports of benznidazole treatment combined with immunosuppression impacting neoplasm development, juxtaposed with instances of CD reactivation successfully treated with benznidazole without concomitant tumor formation (Andrade et al., 2003).

## 2 Discussion and conclusion

Cancer is not solely a genetic ailment; it also carries environmental influences that can either safeguard against malignancy or promote it (Hausman, 2019).

The association between *T. cruzi*, Chagas disease (CD), and cancer presents an unresolved paradox. It is evident that both the parasite, as well as parasite-derived molecules, and the corresponding antiparasitic response can induce modifications in various host cell pathways, leading to alterations in the cell cycle, metabolism, glycosylation, DNA mutations, and neuronal signaling. Furthermore, the presence of the parasite has been demonstrated to induce cell death or a senescent phenotype, influence the immune system, impact the metastatic cascade, and contribute to the formation of new blood vessels.

The contradictory findings in various experimental investigations appear to be, at least in part, related to the existence of different parasite strains. Considerable disparities among strains are evident in terms of phenotypic traits, including pathogenicity, virulence, tissue tropism, the antiparasitic immune response, surface polysaccharides, protein, RNA and DNA content, cytochrome composition, neuroamidase activity, as well as genetic characteristics such as chromosome number, size, gene repertoire, and base composition of nuclear and kinetoplast DNA.

Furthermore, the outcome of *T. cruzi* infection varies depending on the type of tumor analyzed. Tumor cells can influence the parasite, and the antitumor effect notably hinges on factors such as cancer subtype, stage, and the expression of tumor-associated antigens (Ags) and their immunogenicity.

Therefore, based on the extensive discussion in this review and other sources, it can be inferred that the balance of evidence tilts towards the inhibition of tumor growth or resistance to tumor development. While further research is imperative, this is likely applicable to CD patients as well. Moreover, there is a discernible selective or specific component in this effect, typically directed at malignant cells rather than normal ones, encompassing cytotoxins with a preference for neoplastic cells, an ability to preferentially infect tumor cells, and the presence of shared Ags. Nevertheless, non-specific bystander mechanisms, such as the adjuvancy of *T. cruzi* trypomastigotes, or the influence of proinflammatory cytokines like IFN-γ, may also hold significance.

It is our contention that a single molecule or mechanism, as proposed for TcCalr, is insufficient to account for the diversity of outcomes observed and described in this review. Consequently, the myriad effects observed in different models, encompassing *in vivo* and *in vitro* experiments, diverse host organisms and strains, parasite strains, infection or immunization with epimastigote and trypomastigote extracts, and more, are likely the result of multimodal antitumor mechanisms that may operate concurrently or independently.

Therefore, future research efforts should be aimed at achieving a more precise understanding of the molecular and cellular mechanisms at play and identifying the responsible molecules and pathways, with the goal of translating this antitumor potential into innovative therapeutic strategies for cancer treatment. Furthermore, the interaction between the presence of a tumor and the development of infection merits increased attention, as this mutual antagonism may unveil additional insights into the basis of CD (Sheklakova et al., 2003).
